# Finding Needles in a Haystack: Application of Network Analysis and Target Enrichment Studies for the Identification of Potential Anti-Diabetic Phytochemicals

**DOI:** 10.1371/journal.pone.0112911

**Published:** 2014-11-14

**Authors:** Shaik M. Fayaz, Valsala S. Suvanish Kumar, Krishnamurthy G. Rajanikant

**Affiliations:** School of Biotechnology, National Institute of Technology Calicut, Calicut 673601, India; Indian Institute of Integrative Medicine, India

## Abstract

Diabetes mellitus is a debilitating metabolic disorder and remains a significant threat to public health. Herbal medicines have been proven to be effective anti-diabetic agents compared to synthetic drugs in terms of side effects. However, the complexity in their chemical constituents and mechanism of action, hinder the effort to discover novel anti-diabetic drugs. Hence, understanding the biological and chemical basis of pharmacological action of phytochemicals is essential for the discovery of potential anti-diabetic drugs. Identifying important active compounds, their protein targets and the pathways involved in diabetes would serve this purpose. In this context, the present study was aimed at exploring the mechanism of action of anti-diabetic plants phytochemicals through network and chemical-based approaches. This study also involves a focused and constructive strategy for preparing new effective anti-diabetic formulations. Further, a protocol for target enrichment was proposed, to identify novel protein targets for important active compounds. Therefore, the successive use of network analysis combined with target enrichment studies would accelerate the discovery of potential anti-diabetic phytochemicals.

## Introduction

Diabetes mellitus is a major chronic metabolic disorder and an extremely serious condition from both clinical and public health standpoints. Nearly 5% of the world’s population is affected by diabetes. According to the World Health Organisation (WHO) projections, the diabetic population is likely to increase to 300 million or more by the year 2025 [1]. Current studies in India indicate that there is an alarming rise in the prevalence of diabetes, which has gone beyond epidemic to pandemic proportions [2]. Diabetes imperils public health through various complications such as retinopathy, neuropathy, nephropathy, ischemic heart disease, stroke and peripheral vascular disease [3]. Despite the advances in medicinal science, diabetes remains a burning health issue worldwide. Even though the insulin therapy and oral hypoglycemic agents are the first line of treatment for the disease, they show side effects and fail to significantly alter the course of diabetic complications. Due to these reasons, there is a growing interest in herbal medicines [4].

Since time immemorial, herbal remedies have been the highly esteemed source of therapeutics for various diseases. These remedies show great value in treating and preventing diseases, and are currently becoming more mainstream in clinical practice. Herbal medicines are frequently considered to be less toxic and possess fewer side-effects than synthetic drugs [5]. India is sometimes referred to as the botanical garden of the world because it is the largest producer of medicinal herbs and is endowed with a wide diversity of agro-climatic conditions. In India, a number of plants are mentioned in the ancient literature of Ayurveda and Siddha for the treatment of diabetic conditions. Indigenous remedies have been used in India for the treatment of diabetes mellitus since the time of Charaka and Sushruta [6]. Many Indian plants have been investigated for their beneficial use in different types of diabetes [7]. A number of medicinal plants and their polyherbal formulations are being used for treating diabetes in Ayurveda, Siddha and ethnomedicinal practices [8]–[10]. In the traditional system of Indian medicinal plant formulations, combined extracts of plants are used as the drug of choice rather than individual plant extracts [11]. It is believed that the synergistic effect of combined extracts of many plants is more beneficial than extract of a single plant. The inherent anti-diabetic property of these plants is due to their phytochemicals.

Phytochemicals have been the single most prolific source of leads for the development of new drug entities from the dawn of drug discovery. They cover a wide range of therapeutic indications with a great diversity of chemical structures. A number of pure compounds from plant sources were reported to show a blood glucose lowering effect [12]. However, due to extreme complexity both in chemical components and mechanisms of action, the proper use of phytochemicals is still a challenging task and further hinders the effort to design novel anti-diabetic drugs, using the therapeutic principles of herbal medicines. Knowing the biological and chemical basis of pharmacological properties of phytochemicals is important for the identification of novel anti-diabetic drugs. Therefore, due to their extensive use and the therapeutic effects, there is an increasing interest and need to rigorously evaluate the mechanisms of action of herbal products.

This problem could be partially alleviated by the application of comprehensive and advanced computational tools, which might help us to understand the biological and chemical basis of the pharmacological action of phytomedicines. The biological basis of pharmacological action depends on the protein targets on which the phytochemicals act and the pathways involved. The chemical basis of pharmacological action depends on the structural scaffolds of the active compounds. Therefore, structure-based computational approach would help in understanding the critical components responsible for the anti-diabetic activity of the active compounds. Thus, sophisticated computational methods enable us to investigate the complex mechanism of action of drugs and circumvent the challenges associated with biological experiments.

In the present study, we have utilized an integrated network analysis approach of systems biology and chemical analysis approach of structural biology to predict the mechanism of action of anti-diabetic plants and their active compounds. This study would facilitate the understanding of two important aspects. The first one is that since a number of anti-diabetic plants are available, it becomes difficult to identify which among them are important for preparing effective anti-diabetic formulations. In this study, we have tried to address this issue through network analysis of anti-diabetic plants, active compounds, their protein targets and pathways. However, these plants contain a lot of active compounds and they in turn target many proteins and pathways. Therefore, identification of important anti-diabetic plants becomes a daunting task. In this regard, we are proposing the following hypotheses to identify the important anti-diabetic plants, active compounds and protein targets.

### Hypothesis 1

The anti-diabetic plants with maximum number of active compounds would have good anti-diabetic property.

### Hypothesis 2

The active compounds that are present in many anti-diabetic plants may be therapeutically important.

### Hypothesis 3

The active compounds that target many proteins have therapeutic relevancy.

### Hypothesis 4

The proteins that are targeted by many active compounds are important.

### Hypothesis 5

The protein targets that are involved in multiple pathways related to diabetes are important.

The present study was designed to examine these hypotheses. In order to infer hypothesis 1 and 2, a network containing anti-diabetic plants and active compounds was built and analyzed. To infer hypothesis 3 and 4, a network containing active compounds and protein targets was built and analyzed. Further, to infer hypothesis 5, a network containing protein targets and pathways was built and analyzed. Therefore, through network analysis, inferences were made for these hypotheses. These inferences would assist in the identification of important active compounds and thus the anti-diabetic plants containing these compounds could be used for preparing anti-diabetic formulations. Further, by conceptualizing this entire analysis, a strategy for preparing new anti-diabetic formulations has been proposed in this study.

The second aspect is to identify the biological and chemical basis of pharmacological action of phytochemicals. This was achieved through network analysis of phytochemicals and their respective protein targets and pathways involved in diabetes. Further, pharmacophore analysis of the phytochemicals, combined with docking studies, provided insight in to their mechanism of action.

**Figure 1:**
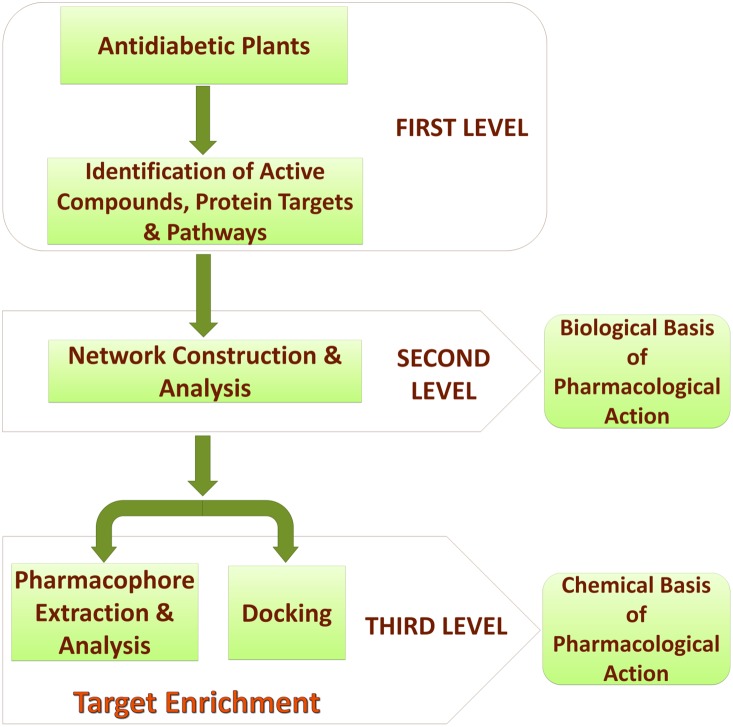
The three levels of methodology used in this study. First level involves identification of active compounds, protein targets and pathways. Second level involves network construction and analysis. This helps in the identification of the biological basis of pharmacological action of anti-diabetic plants. Third level involves target enrichment through pharmacophore analysis and docking studies. This helps in the identification of the chemical basis of pharmacological action of anti-diabetic plants.

This entire study is conceptualized into three levels (Figure 1). The first level is the selection of anti-diabetic medicinal plants of Indian origin, identification of their active compounds, protein targets and pathways from the available literature and existing databases. The second level involves construction and analysis of meaningful networks from the collected data. In the third and the final level, target-enrichment was carried out to enhance the set of multi-targeting active compounds through pharmacophore feature analysis, structural similarity and docking studies. Pharmacological promiscuous bioactive compounds are gaining increased attention in the field of drug discovery. In this regard, the current target-enrichment approach would extend the path for the identification of novel multi-targeting bio-active molecules, deciding the fate of various diabetes-related pathways and thus would aid in the treatment of diabetes.

## Materials and Methods

### 1. Data collection

An extensive literature survey was carried out in this regard. The anti-diabetic plants available in India were selected for this study (Table 1). Almost all the plants [except ADP14] are being used in Ayurvedic treatment (Table 1).

**Table 1:** Anti-diabetic plants (Scientific and Sanskrit names) and their respective codes.

SL No.	Anti-diabetic Plant[Sanskrit name]	Code	Reference	SL No.	Anti-diabetic Plant[Sanskrit name]	Code	Reference
1	*Citrus maxima* (Burm.)Merr. [Madhukarkati]	ADP1	[85]	16	*Phyllanthus niruri var.* *amarus* (Schumach. &Thonn.) Leandri [Bhumyamalaki]	ADP16	[98]
2	*Morus alba* L.[Tuda/Tooda]	ADP2	[86]	17	*Cullen corylifolium* (L.)Medik. [Bakuchi]	ADP17	[99]
3	*Abelmoschus moschatus*Medik. [latakasturikam]	ADP3	[87]	18	*Salacia oblonga* Wall.[Saptarangi]	ADP18	[100]
4	*Allium sativum* L.[Lasuna]	ADP4	[88]	19	*Sesamum orientale* L.[Snehaphala]	ADP19	[101]
5	*Cyamopsis tetragonoloba*(L.) Taub. [Bakuchi]	ADP5	[89]	20	*Swertia chirayita*(Roxb. ex Fleming)H. Karst[Anaryatikta]	ADP20	[91]
6	*Hydnocarpus wightiana*Blume. [Tuvaraka]	ADP6	[90]	21	*Syzygium cumini* (L.)Skeels [Jambu]	ADP21	[102]
7	*Salacia reticulate* Wight.[Vairi]	ADP7	[91]	22	*Vigna mungo var.* *silvestris* Lukoki,Maréchal & Otoul,E. [Mashaparni]	ADP22	[103]
8	*Tinospora malabarica*(Lam.) Hook. f. &Thomson [Gulvel sattva]	ADP8	[91]	23	*Zingiber officinale*Roscoe [Ardraka]	ADP23	[104]
9	*Zanthoxylum armatum*DC. [Tejasvini]	ADP9	[92]	24	*Trigonella foenum-* *graecum* L. [Medhika]	ADP24	[105]
10	*Achyranthes aspera* L.[Apamarga]	ADP10	[93]	25	*Eclipta prostrata* (L.)L. [Bhringaraj]	ADP25	[106]
11	*Cajanus cajan* (L.)Huth [Adhaki]	ADP11	[91]	26	*Aegle marmelos* (L.)Corrêa [Bilva]	ADP26	[107]
12	*Nelumbo nucifera*Gaertn. [Padma]	ADP12	[94]	27	*Syzygium cumini var.* *cumini* [Jambu]	ADP27	[91]
13	*Withania somnifera* (L.)Dunal [Ashwagandha]	ADP13	[95]	28	*Casearia esculenta*Roxb. [Saptchakra]	ADP28	[108]
14	*Perilla frutescens* (L.)Britton [Ajeka]	ADP14	[96]	29	*Avena sativa* L.[Jai/Yavika]	ADP29	[109]
15	*Phaseolus vulgaris* L.[Mudga]	ADP15	[97]				

The active compounds present in these plants were identified through the review of published research articles from PubMed-NCBI and from the databases such as Dr. Duke’s phytochemical and ethnobotanical database, Floridata, PhytoDiabCare, Plants for a Future database, DIACAN and DADMP (Table 2). During the literature survey, we collected only the compounds that were reported to be active compounds or that have shown anti-diabetic activity.

**Table 2:** Active compounds and their respective codes.

SL No.	Active Compound	Code	SL No.	Active Compound	Code
1	Synephrine	AC1	30	Perilloside C	AC33
2	1-deoxynojirimycin	AC2	31	Beta estradiol	AC34
3	Betulinic acid	AC3	32	Kaempferol-3-o-glucoside	AC35
4	Beta sitosterol	AC4	33	Delphinidin	AC36
5	Oleanolic acid	AC5	34	Gallocatechin	AC37
6	Myricetin	AC6	35	Angelicin	AC38
7	Kaempferol	AC7	36	Bakuchicin	AC39
8	Apigenin	AC9	37	Corylifolin	AC40
9	Ferulic acid	AC10	38	Bakuchiol	AC41
10	Gallic acid	AC11	39	Diadzein	AC42
11	Ellagic acid	AC12	40	Stigmasterol	AC43
12	Astragalin	AC13	41	Pinoresinol	AC44
13	Quercetin	AC14	42	Arachidonic acid	AC45
14	Genistein	AC15	43	Folic acid	AC46
15	Luteolin	AC16	44	Guaiacol	AC47
16	Mangiferin	AC17	45	Oleic acid	AC48
17	Salacinol	AC18	46	Coniferin	AC49
18	Kotalanol	AC19	47	Palmitic acid	AC50
19	(−)-Epigallocatechin	AC20	48	Stearic acid	AC51
20	(−)-Epicatechin-(4beta–>8)-(−)-4′-O-methylepigallocatechin	AC21	49	Linolenic acid	AC52
21	(−)-Epicatechin	AC22	50	Pipecolic acid	AC53
22	3-beta dihydroxyolean-12-en-29-oic acid	AC23	51	Shogaol	AC54
23	22-beta dihydroxyolean-12-en-29-oic acid	AC24	52	Geraniol	AC55
24	Berberine	AC25	53	Isoquercitrin	AC56
25	Avenasterol	AC26	54	Diosgenin	AC57
26	Amyrin	AC27	55	Wedelolactone	AC58
27	Protocatechuic acid	AC30	56	Marmesin	AC59
28	osmarinic acid	AC31	57	Beta glucan	AC60
29	Perilloside A	AC32			

The proteins that are targeted by the active compounds were identified from research publications (PubMed-NCBI) and also from multiple online databases like DrugBank, Therapeutic Target Database (TTD), PharmGKB, STITCH and SuperTarget (Table 3). The protein target collection was focused on the mammalian system of biochemical processes, to suit the study. All the protein targets that are directly (through binding of active compound) or indirectly influenced by the active compounds were collected. This data was used to build networks. Further, the dataset was filtered on the basis of direct interaction between the active compound and the protein target and only these direct interactions were carried down for the target enrichment studies.

**Table 3:** Protein targets and their respective codes.

SL No.	Protein Target	Code	SL No.	Protein Target	Code
1	Mono amine oxidase	PT1	34	Phospholipase A2	PT41
2	Glucosidase	PT2	35	Glutamate dehydrogenase	PT42
3	Alpha glucosidase	PT3	36	Glutathione transferase	PT43
4	Glycogen debranching enzyme	PT5	37	Pyruvate dehydrogenase	PT44
5	Cytosolic beta glucosidase	PT6	38	Protein tyrosine phosphatase	PT45
6	Lactase glycosylceramidase	PT7	39	Thromboxane synthase	PT46
7	Glucosidase II beta subunit precursor	PT8	40	IKK beta	PT47
8	Diacylglycerol acyltransferase	PT9	41	Acetylcholine esterase	PT48
9	Nitric oxide synthase	PT10	42	IGF	PT49
10	5-alpha reductase	PT11	43	NADPH oxidase	PT50
11	Glycogen phosphorylase	PT12	44	Insulin receptor	PT51
12	Gaba transaminase	PT13	45	PI3K	PT52
13	Alpha amylase	PT14	46	Lipoxygenase	PT53
14	Xanthine oxidase	PT15	47	Maltase glucoamylase	PT54
15	Aldose reductase	PT16	48	Beta secretase1 (BACE1)	PT55
16	20-alpha hydroxysteroid dehydrogenase	PT21	49	NFK-beta	PT56
17	Carbonic anhydrase	PT22	50	PSORS1C2	PT57
18	Udp glucose dehydrogenase	PT23	51	Lysosomal alpha glucosidase precursor	PT58
19	Ribonucleotide reductase	PT24	52	Tyrosine phosphatase 1B	PT59
20	COX 1	PT25	53	LXR alpha	PT60
21	COX 2	PT26	54	DNA topoisomerase 1	PT61
22	Steroid-5-alpha reductasetype II	PT27	55	Protein tyrosine phosphatase 1B	PT62
23	Pancreatic lipase	PT29	56	NA+/K+ -ATPase	PT63
24	Lipoprotein lipase	PT30	57	3-Beta hydroxysteroid dehydrogenase type II	PT64
25	AMPK	PT31	58	Quinone reductase 2	PT65
26	Caspase 3	PT32	59	CARM1	PT66
27	Catechol-o-methyltransferase	PT34	60	Protein tyrosine kinase	PT67
28	Estrogen receptor	PT35	61	IGFR	PT68
29	MAPK	PT36	62	Sucrase isomaltasen-terminal domains	PT69
30	Glyoxalase	PT37	63	7-Dehydrocholesterol reductase	PT70
31	DNA topoisomerase 2	PT38	64	Protein tyrosine kinase IIB	PT71
32	DNA Polymerase	PT39	65	FABP4	PT72
33	DNA Polymerase beta	PT40			

The biological pathways of the respective protein targets and the diseases in which they are involved were retrieved from databases such as BRENDA, KEGG, PDTD, Binding DB, Pathway Interaction Database, WikiPathways, GeneCards, SMPDB, Reactome, Pathway Linker and SPIKE (Table 4).

**Table 4:** Diabetes-related pathways and their respective codes.

SL No.	Pathways	Code	SL No.	Pathways	Code
1	Aminoacid metabolism	P1	23	Aldosterone-regulated sodium reabsorption	P26
2	Glycogen degradation	P2	24	Pyruvate metabolism	P27
3	N-Glycan biosynthesis	P3	25	Cell cycle and DNA replication	P28
4	Starch and sucrose metabolism	P4	26	Alpha-linolenic acid metabolism	P29
5	Galactose metabolism	P5	27	Linoleic acid metabolism	P30
6	Glycerolipid metabolism	P6	28	Glutamine biosynthesis	P31
7	Apoptosis	P7	29	Glutathione metabolism	P32
8	Steroid hormone biosynthesis	P8	30	Metabolism of xenobiotics by CYP450	P33
9	Starch degradation-I	P9	31	Drug metabolism by CYP450	P34
10	Carbohydrate digestion and absorption	P10	32	Citrate cycle (TCA cycle)	P35
11	Pancreatic secretion	P11	33	JAK-STAT cascade in growth hormone signaling pathway	P36
12	Purine metabolism	P12	34	MAPK signaling pathway	P37
13	Fructose and mannose metabolism	P13	35	Chemokine signaling pathway	P38
14	Glycolysis/Gluconeogenesis	P14	36	NFKB activation	P40
15	Nitrogen metabolism	P18	37	Leukocyte endothelial migration	P41
16	Arachidonic acid metabolism	P19	38	Osteoclast differentiation	P42
17	Sucrose metabolic process	P20	39	ROS generation and oxidative stress	P43
18	Retinoid metabolism and transport	P21	40	Adherens junction	P44
19	PPAR signaling pathway	P22	41	Inositol phosphate metabolism	P45
20	Insulin signaling pathway	P23	42	Phosphatidylinositol signaling pathway	P46
21	BDNF signaling pathway	P24	43	JAG1-NOTCH pathway	P47
22	STAT3 signaling pathway	P25	44	Toll-like receptor signaling pathway	P48

### 2. Network construction and analysis

In order to find the biological means of pharmacological action of anti-diabetic plants, networks were constructed through Cytoscape v3 software [13]. Using alternative combinations of anti-diabetic plants (ADP), active compounds (AC), protein targets (PT), biochemical pathways (P) and diseases, the networks ADP-AC, AC-PT, PT-P and PT-Diseases were constructed, respectively. The networks thus created were analyzed for potential nodes and hubs.

### 3. Structures of proteins and active compounds

The information regarding these active compounds and their structures were collected from PubChem. The structure for each protein target was retrieved from protein data bank (PDB). If multiple protein structure entries were available in PDB, then the structure solved through X-ray diffraction and also with good resolution was considered.

### 4. ADME prediction

The ADME (absorption, distribution, metabolism, and excretion) properties of the active compounds were studied using the QikProp program in Schrodinger suite (Schrödinger LLC, New York, NY). QikProp efficiently predicts and evaluates physically significant descriptors and pharmaceutically relevant properties of molecules, making it an indispensable tool for applying ADME principles in lead discovery and optimization.

The properties of all the compounds were predicted by processing the program in normal mode. Principal descriptors and physiochemical properties with a detailed analysis of the log P (Octanol/Water) and % human oral absorption were predicted. The acceptability of the compounds based on Lipinski’s rule of 5 [14], which predicts the drug likeliness essential for rational drug design, was also evaluated.

### 5. Structural alignment and pharmacophore extraction

The active compounds that have a common protein target were used for pharmacophore generation. The 3D structures of active compounds were prepared using the LigPrep module (Schrödinger LLC). In this step, all the possible conformations of the compounds were generated. The conformations of all the active compounds for a particular protein were aligned and the pharmacophores were extracted. The structural alignment of compounds and the pharmacophore extraction was carried out using the Phase module (Schrödinger LLC). The pharmacophores thus extracted contained six features: H-bond acceptor (A), H-bond donor (D), hydrophobic group (H), negatively ionizable (N), positively ionizable region (P), and aromatic ring (R). These features are necessary for the active compounds to bind to their protein targets.

### 6. Docking studies

The protein structures extracted from PDB were initially prepared using the protein preparation wizard. In this step, the structures were treated to add missing hydrogens, assign proper bond orders and delete unwanted water molecules, ligands and protein chains. The H-bonds were optimized using sample orientations. Finally, the protein structures were minimized to the default Root Mean Square Deviation (RMSD) value of 0.30 A^o^. Glide energy grids were generated for each of the prepared proteins. A grid box of approximately 10 A^o^ was around the binding site of the protein. The atoms were scaled by van der Waals radii of 1.0 A^o^ with the partial atomic charge less than 0.25 defaults. No constraints were defined. These grids were employed in the docking studies.

Docking of active compounds to their respective protein targets was carried out using the Glide module (Schrödinger LLC) [15]. The XP (Extra-Precision) docking method in glide was used to dock the compounds in to the binding site of the protein, using OPLS 2005 force field. The default settings as available in the software package were used for the refinement and docking calculations. The van der Waals radii were scaled using a default scaling factor of 0.80 and default partial cut-off charge of 0.15 to decrease the penalties. The option to output Glide XP descriptor information was chosen, which deduces energy terms such as H-bond interactions, electrostatic interaction, hydrophobic enclosure, and π-π stacking interactions. The binding modes and interactions of active compounds with their respective protein targets were analyzed from the docking output.

## Results

### 1. Anti-diabetic Plants (ADP) - Active Compounds (AC) network

**Figure 2:**
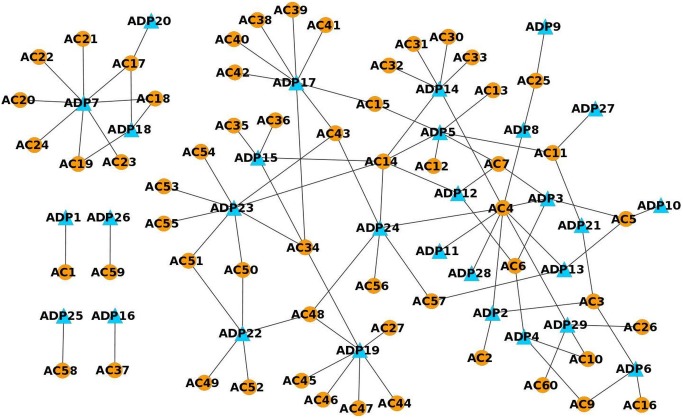
Network containing anti-diabetic plants and their respective active compounds.

The active compounds present in all the anti-diabetic plants were collected and a network was built from this data (Figure 2). The anti-diabetic plants ADP5, ADP7, ADP14, ADP17, ADP19, ADP22, ADP23 and ADP24 contain many active compounds. These plants cover nearly 70% of the entire active compounds. Since the anti-diabetic property of the plant is due to the plant’s active compounds, it may be hypothesized that a plant with the maximum number of active compounds would have good anti-diabetic property (Hypothesis 1).

The active compounds AC3, AC4, AC5, AC6, AC7, AC11, AC14, AC17, AC34, AC43 and AC48 are present in many anti-diabetic plants. Since these compounds are present in many plants, they may be important for anti-diabetic activity. Hence, it could be hypothesized that the active compounds that are present in many anti-diabetic plants may be therapeutically important (Hypothesis 2).

Presence of common active compounds in plants shows that the anti-diabetic activity of these plants might be due to common mechanisms. However, the mechanism of action of these active compounds that are involved in anti-diabetic activity need to be unravelled. This could be achieved by studying the proteins that are targeted by these compounds.

### 2. Active Compounds (AC) - Protein Targets (PT) network

**Figure 3:**
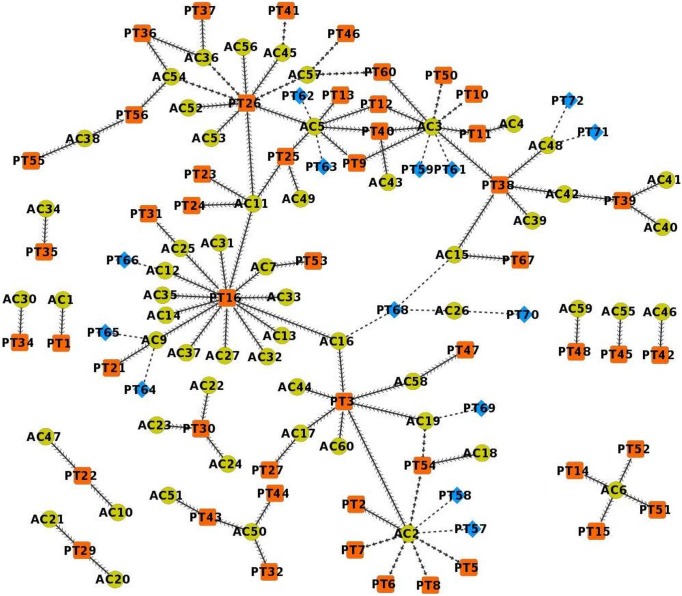
Network containing active compounds and their respective protein targets. Contiguous arrow – AC directly targets PT involved in diabetes, Separate arrow – AC indirectly targets PT involved in diabetes, Dash line – AC targets PT not involved in diabetes.

The anti-diabetic property of plants depends on their active compounds, which in turn relies upon the proteins that are targeted by them. It means that the active compounds that target proteins involved in diabetes will have anti-diabetic activity. In this regard, we have collected all the proteins that are targeted by these active compounds. The network built from this data shows active compounds and their protein targets that either are or are not involved in diabetes (Figure 3). When the protein targets involved in diabetes are considered, the active compounds may be targeting them directly by binding to them, or indirectly by influencing other proteins.

Among the proteins that are targeted by the active compounds, most of them are involved in diabetes. The active compounds AC2, AC3, AC5, AC6 and AC11 target either directly or indirectly many proteins involved in diabetes. This proves the known fact that the action of phytochemicals is typically multi-targeted. A single active compound may target multiple proteins involved in a particular disease. Among the diabetes-related proteins that are targeted by these compounds, the majority are exclusive to them and are not shared by any other compound. Here, it may be hypothesized that the active compounds that target many proteins have therapeutic relevancy (Hypothesis 3). AC3, AC5, AC6 and AC11 are present in many anti-diabetic plants, as listed in the previous section, whereas AC2 is present only in ADP2.

The protein targets PT3, PT16, PT25, PT26, PT30, PT38, PT39, PT40, and PT54, which are involved in diabetes, are targeted by many active compounds. Hence, it may be hypothesized that these proteins are important since they are targeted by most of the active compounds (Hypothesis 4).

Interestingly, most of the proteins that are targeted by many compounds are covered by the active compounds that target many proteins. Presence of common active compounds in anti-diabetic plants and in turn the presence of common proteins that are targeted by most of the active compounds indicate that the plants and compounds share common mechanism of action, which imparts them anti-diabetic property.

### 3. Protein Targets (PT) – Pathways (P) Network

**Figure 4:**
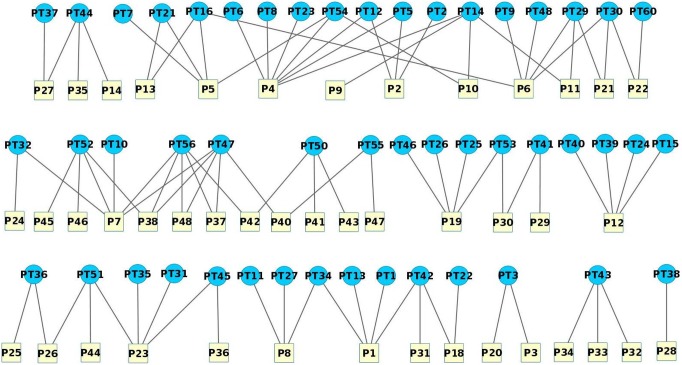
Network containing protein targets and their respective pathways.

The pathways with which the protein targets related to diabetes are involved were collected and a network was constructed (Figure 4). The pathways P1, P4, P5, P6, P7, P12, P19 and P23 involve many protein targets. The protein targets PT14, PT47, PT52 and PT56 are involved in many pathways. These proteins are important since their modulation would alter multiple pathways related to diabetes. Interestingly, none of these protein targets match those that are targeted by many active compounds. Thus, it may be hypothesized that the proteins that are involved in multiple pathways related to diabetes are important (Hypothesis 5).

### 4. Protein Targets – Diseases Network

**Figure 5:**
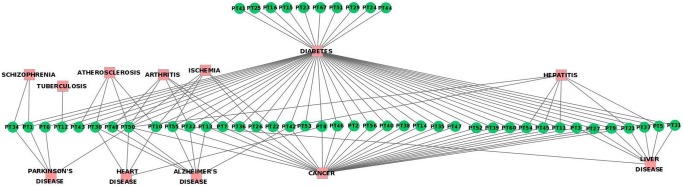
Network containing various diseases in which the protein targets are involved.

The data regarding different diseases in which the diabetes-related protein targets are involved was collected and a network was drawn from it (Figure 5). Here it can be seen that most of the proteins involved in diabetes are also involved in cancer. Diabetes and cancer are two heterogeneous, multifactorial, severe, and chronic diseases. Numerous studies have shown a contiguous association between diabetes and cancer. The American Diabetes Association and the American Cancer Society jointly stated that cancer incidence is associated with diabetes as well as certain diabetes risk factors and diabetes treatments [16]. The association between diabetes and cancer incidence or prognosis and existence of risk factors common to both the diseases has been reported [16]. Further, epidemiological studies have indicated that the risk of several types of cancer (including pancreas, liver, breast, colorectal, urinary tract, and female reproductive organs) has increased in diabetic patients [17].

Apart from cancer, many proteins are involved in other diseases like Parkinson’s disease, Alzheimer’s disease, ischemia, hepatitis and liver diseases. It was reported that type 2 diabetes (T2D) is associated with an increased risk of Parkinson’s disease [18]. Numerous studies have shown that common pathophysiological mechanisms exist between Parkinson’s disease and T2D [19]. Further, an integrative network analysis has unveiled convergent molecular pathways in Parkinson’s disease and diabetes [20]. Mounting evidence also indicates the shared mechanisms of pathogenesis between T2D and Alzheimer’s disease [21]. Alzheimer’s disease represents a form of diabetes that selectively involves the brain. It also contains molecular and biochemical features that overlap with both T1D and T2D. Therefore, Alzheimer’s disease was termed as type 3 diabetes [22]. Further, intranasal administration of insulin in patients with Alzheimer’s disease exhibited neuroprotective effects [23], [24]. In diabetic patients, cardiovascular disease remains the leading cause of death. Myocardial infarctions and ischemia tend to be more extensive and have a poorer survival rate compared to individuals without diabetes. Similarly, liver diseases were also reported to be very well associated with diabetes [25].

**Figure 6:**
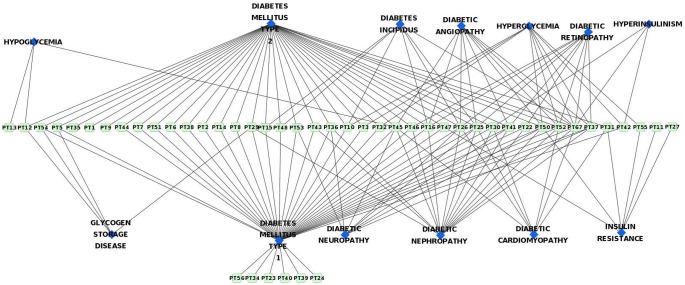
Network containing protein targets involved in different diabetic complications.

The relation between protein targets and different types of diabetes and its related complications has been studied and the network constructed from this data shows that the proteins are mainly involved in T1D, T2D, diabetic neuropathy, diabetic nephropathy, diabetic angiopathy, diabetic retinopathy and hyperglycemia (Figure 6). Most of the proteins involved in T1D are also involved in T2D and vice versa. This may be due to the similar pathophysiological mechanisms involved in both the types of diabetes. For instance, T1D and T2D are characterized by progressive beta-cell failure. Apoptosis is probably the main form of beta-cell death in both forms of the disease. The mechanisms leading to nutrient- and cytokine-induced beta-cell death in T2D and T1D, respectively, share the activation of a final common pathway involving interleukin IL-1beta, nuclear factor NF-kappaB, and Fas. All forms of diabetes increase the risk of long-term complications. The major long-term complications are related to the damage of blood vessels and are known as microvascular diseases. The primary microvascular complications of diabetes include damage to the eyes, kidneys, and nerves, known as diabetic retinopathy, diabetic nephropathy and diabetic neuropathy, respectively. From Figure 6, it is evident that these diabetic complications share similar protein targets and hence similar pathological mechanisms.

### 5. Hypotheses and inferences

**Figure 7:**
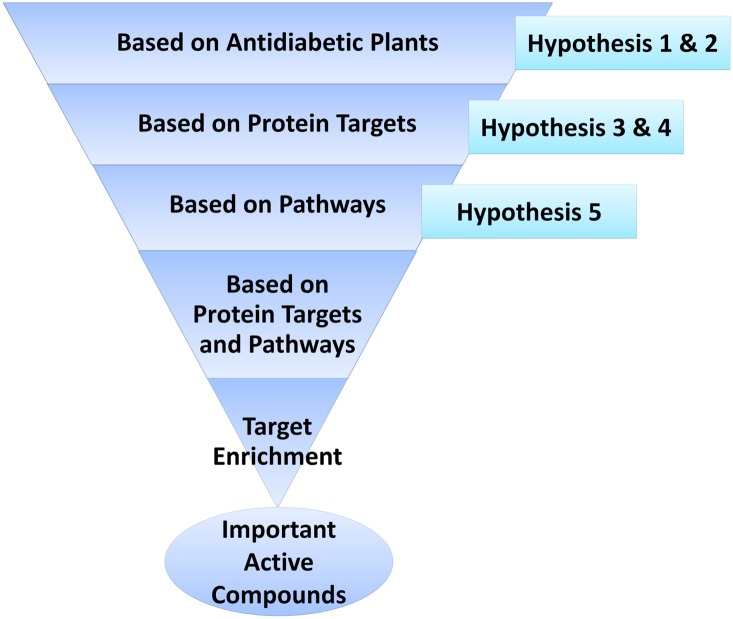
Step-wise representation of the discovery of important/potential active compounds that have been discovered, and the various hypotheses made during these stages.

In this study, different hypotheses were made at various stages (Figure 7). Inferences were made through careful observation of various networks.

#### Hypothesis 1

The anti-diabetic plants with the maximum number of active compounds would have good anti-diabetic property.

#### Inference

This might not be true. The anti-diabetic property of plants depends on the mechanism of action of their active compounds. The anti-diabetic plant that contains therapeutically relevant active compounds need to be considered important, rather than the one with maximum number of active compounds. For example, ADP7, ADP17 and ADP19 contain many active compounds (Figure 2). However, these compounds target very few proteins or pathways involved in diabetes (Figure 3). Hence, these plants might not exhibit comparatively good anti-diabetic property and thus need not be considered important for anti-diabetic treatment. Further, it was observed that most of the important active compounds, like AC2, AC3, AC6, AC11 and AC58 that target multiple proteins and pathways closely related to diabetes, are present in plants (ADP2, ADP21, ADP4, ADP21, ADP27 and ADP25 respectively) that do not contain many active compounds (Figure 2).

#### Hypothesis 2

The active compounds that are present in many anti-diabetic plants may be therapeutically important.

#### Inference

This might not be true. For example, AC4 present in the maximum number of anti-diabetic plants, targets only one protein target (PT11), which is involved in only one pathway (Figures 2–4). Similarly, the compounds AC7, AC14, AC17, AC34, AC43 and AC48 that are present in many anti-diabetic plants target very few proteins and pathways. On the contrary, the active compounds, AC2, AC54 and AC58 that target multiple proteins and pathways are present only in ADP2, ADP23, and ADP25, respectively (Figure 2). However, the active compounds AC3, AC5 and AC11 that also target multiple proteins and pathways are present in many plants. But the importance of the active compound depends only on its mechanism of action and not on the number of plants in which it is present.

#### Hypothesis 3

The active compounds that target many proteins have therapeutic relevancy.

#### Inference

This is true. Since proteins play a key role in the pathways and disease pathology, they need to be modulated. Diabetes is a multifactorial disease that involves various proteins, pathways and mechanisms. Targeting multiple disease-related proteins and pathways would be beneficial in such diseases. For instance, AC2, AC3, AC5, AC6 and AC11 target multiple proteins involved in diabetes (Figure 3). Hence, due to this polypharmacological effect, they might exhibit better anti-diabetic activity compared to other compounds. Therefore, active compounds that target multiple proteins involved in the disease-related mechanisms are important.

#### Hypothesis 4

The proteins that are targeted by many active compounds are important.

#### Inference

This might not be true. For instance, the proteins PT3, PT16 and PT26 are targeted by many active compounds (Figure 3). However, these targets are not involved in multiple pathways related to diabetes. Thus, the proteins with higher involvement in the disease pathology need to be considered important rather than those that are targeted by multiple active compounds.

#### Hypothesis 5

The protein targets that are involved in multiple pathways related to diabetes are important.

#### Inference

This is true. The protein targets that are involved in multiple pathways related to diabetes are the proteins with higher involvement in the disease pathology. Therefore, they need to be targeted for effective disease treatment. For instance, the proteins PT14 and PT47 are involved in multiple pathways related to diabetes (Figure 4). Hence, targeting these proteins would prove to be beneficial rather than targeting any of the proteins involved in diabetes.

Thus, these hypotheses and inferences revealed the key phytochemicals and protein targets involved in diabetes. This would help in the preparation of effective anti-diabetic formulations that contain therapeutically important active compounds.

### 6. Strategy for formulations

Through network analysis, the active compounds present in anti-diabetic plants, their protein targets and the pathways involving these targets have been studied. This would provide an overall view of the mechanism of action of anti-diabetic plants. Further, this would help us to know which proteins need to be targeted and hence, which anti-diabetic plants or rather which active compounds need to be used to achieve this. Therefore, this type of study would assist us in preparing new effective anti-diabetic formulations. In fact, the present strategy could be used to prepare more focused and goal oriented formulations for any disease.

Polyherbal formulations with various active principles and properties have been used from ancient days to treat a whole range of human diseases. Generally, they are a collection of therapeutic recipes that are formulated and prepared on the basis of the healing properties of individual ingredients with respect to the condition of sickness. Such herbal constituents with diverse pharmacological actions principally work together in a dynamic way to produce maximum therapeutic efficacy with minimum side effects. Therefore, studying the biological means of action of various medicinal plants for a disease and then preparing the formulation based on this information would be a more constructive and focused approach. Here, considering plant extracts that contain active compounds that target multiple therapeutically relevant protein targets would be an ideal choice.

For anti-diabetic treatment, we need to consider and target most of the disease-related proteins and pathways. Therefore, a good or ideal formulation would contain active compounds that target multiple proteins as well as the proteins that are involved in multiple pathways related to diabetes. Based on the plants considered in this study, an ideal formulation would be the plant extract containing the active compounds AC2, AC3, AC5, AC6, AC11, AC54 and AC58. The plants containing such active compounds should be considered during anti-diabetic formulations.
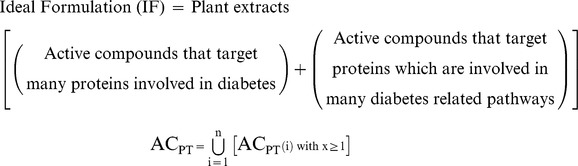



[AC_PT_ = Active compounds that target many proteins involved in diabetes, i.e., combination of all the active compounds with x≥1].
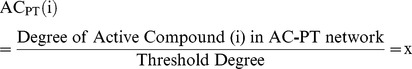



i = 1 to n (n = number of active compounds).

Degree is an important feature in network analysis. The degree of a node ‘N’ is the number of nodes connecting it. If three nodes are connecting N, then the degree of this node is three. Threshold Degree is the minimum number of nodes an active compound should be targeting to consider it important. This is manually suggested by analyzing the minimum and maximum degree of active compounds. For example, in this study, the minimum and maximum degree of AC nodes in the AC-PT network are 1 and 8, respectively. If we consider an optimum value of 4 as threshold degree, then AC_PT_ of AC5 (i.e., i = 5) would be,
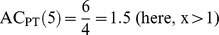



Similarly, when AC_PT_(i) is calculated for all the active compounds, then AC_PT_ would be,
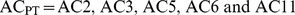



AC_PT_ would help us identify the active compounds that target many proteins. In order to find the active compounds that target proteins which are involved in many pathways,
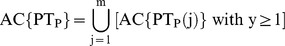
[AC{PT_P_} = Active compounds that target proteins which are involved in many pathways, i.e., combination of all the active compounds that target proteins with y≥1].

[AC{PT_P_(j)} = Active compound with highest degree among all the compounds that target PT_P_(j)].
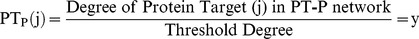



j = 1 to m (m = number of protein targets).

In this study, if AC{PT_P_} is calculated with threshold degree 4, then it would be,
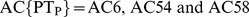



Therefore, Ideal formulation (IF) would be,
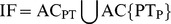



Finally, from our study, IF would contain active compounds AC2, AC3, AC5, AC6, AC11, AC54 and AC58. Thus, they could be considered the important active compounds and the plants containing these compounds might be used for anti-diabetic formulations.

The IF proposed in this study is based on the fact that targeting multiple proteins involved in diabetes is essential for better anti-diabetic activity. However, the active compounds present in the IF might not target all the important proteins involved in diabetes. In order to cover these important proteins, we might have to include additional active compounds other than those reported in the IF. This might ultimately result in a huge list of compounds and their respective plants. However, this number could be reduced by increasing/enriching the protein targets of the active compounds present in IF. This could be achieved by predicting whether these active compounds could bind to additional proteins, other than those reported in the literature, through structural and pharmacophore studies. In this context, we are proposing a novel methodology named target enrichment to predict additional protein targets of the active compounds. This would help in deciding the active compounds that may target all the important proteins and would make them an ideal choice for formulations.

### 7. Target enrichment

Since most of the phytochemicals act on multiple targets and exhibit diverse effects, the active compounds that bind to multiple protein targets would be beneficial. Hence, it would be advantageous if we could add new targets to the known active compounds. Target enrichment acts here precisely by predicting novel protein targets. We have employed target enrichment to further increase or enrich the protein targets of active compounds so that it would help us predict which compound would finally bind to a higher number of proteins. This would in turn help in developing effective anti-diabetic polyherbal formulations.

Target enrichment was performed through pharmacophore analysis and docking studies. A pharmacophore is an abstract description of molecular features, which are necessary for molecular recognition of a ligand by a biological macromolecule like protein. The features such as H-bond acceptor, H-bond donor, aromatic ring and hydrophobic group are represented in a pharmacophore. A pharmacophore model explains how structurally diverse ligands can bind to a common receptor site. Numerous studies have shown that the compounds that share similar pharmacophoric features bind to similar proteins [26]–[28]. This concept has been adopted in our target enrichment study. Since pharmacophore of an active compound represents its binding to a protein target, the target enrichment would only involve active compounds and their ‘directly’ targeted proteins.

**Figure 8:**
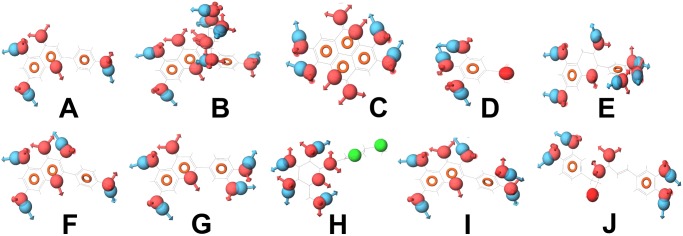
Compounds (with their pharmacophoric features) that target aldose reductase. (A) Apigenin, (B) Astragalin, (C) Ellagic acid, (D) Gallic acid, (E) Gallocatechin, (F) Kaempferol, (G) Luteolin, (H) Perilloside A, (I) Quercetin and (J) Rosmarinic acid. These compounds show similar pharmacophoric features.

**Figure 9:**
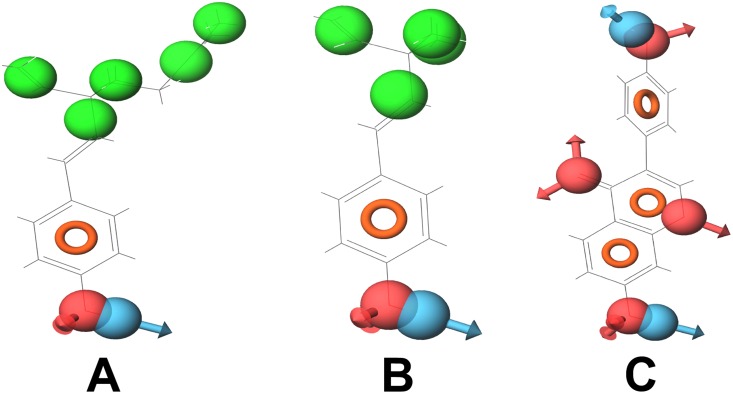
Compounds (with their pharmacophoric features) that target DNA polymerase. (A) Bakuchiol, (B) Corylifolin and (C) Diadzein. These compounds show similar pharmacophoric features.

**Figure 10:**
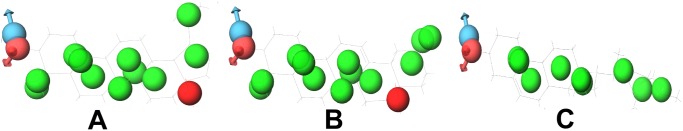
Compounds (with their pharmacophoric features) that target DNA polymerase beta. (A) Betulinic acid, (B) Oleanolic acid and (C) Stgmasterol. These compounds show similar pharmacophoric features.

**Figure 11:**
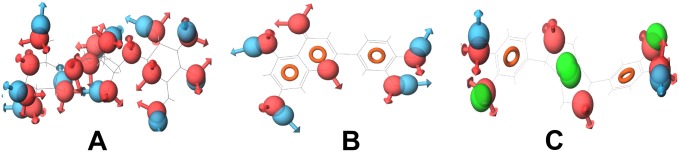
Compounds (with their pharmacophoric features) that target alpha glucosidase. (A) Beta Glucan, (B) Luteolin and (C) Pinoresinol. These compounds show similar pharmacophoric features.

In this study, the pharmacophore analysis showed that the compounds that have similar pharmacophoric features bind to the same target. For example, the active compounds that bind to their respective protein targets like aldose reductase, DNA polymerase, DNA polymerase β and alpha glucosidase have similar pharmacophoric features (Figures 8–11). All the compounds that bind to aldose reductase share similar pharmacophoric features (Figure 8). It can be seen that the benzene rings on the left harbours pharmacophoric features that are common to all of them. The compounds that target DNA polymerase also share similar pharmacophoric features (Figure 9). They contain an aromatic benzene ring to which an H-bond acceptor and a donor group are attached. Similarly, all the compounds of DNA polymerase β share similar pharmacophoric features (Figure 10). On their left side, they contain an H-bond acceptor and a donor group. The compounds of alpha glucosidase do not have similar structures. However, they share few common pharmacophoric features, due to which, all of them might be binding to alpha glucosidase (Figure 11).

**Figure 12:**
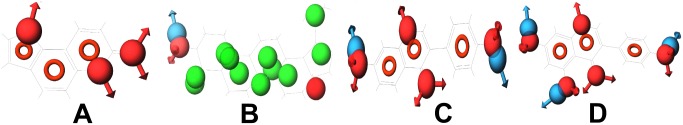
Compounds (with their pharmacophoric features) that target DNA topoisomerase 2. (A) Bakuchicin, (B) Betulinic acid, (C) Diadzein and (D) Genistein. These compounds do not show similar pharmacophoric features. Only diadzein is similar to genistein.

**Figure 13:**
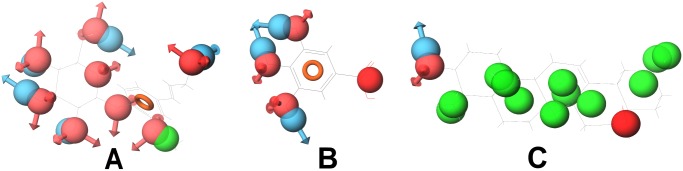
Compounds (with their pharmacophoric features) that target COX-1. (A) Coniferin, (B) Gallic acid and (C) Oleanolic acid. These compounds do not show similar pharmacophoric features. Only coniferin and gallic acid have little structural similarity.

Since the compounds with similar structures and pharmacophoric features bind to similar protein targets, during target enrichment, we would consider active compounds that have similar pharmacophoric features and then append the protein targets of each compound to all of them. However, though structurally similar compounds bind to similar targets, the inverse is not always true. That is, all the compounds that bind to a particular target might not have similar structures and pharmacophoric features. For example, compounds that bind to Cyclooxygenase-1 (COX-1), Cyclooxygenase-2 (COX-2) and DNA topoisomerase 2, respectively, do not have similar structures and pharmacophoric features. Among the compounds of DNA topoisomerase 2, only diadzein is similar to genistein (Figure 12). In the case of COX-1, only coniferin and gallic acid have little structural similarity (Figure 13). This may be due to the fact that the binding of compounds to a protein not only depends on their pharmacophoric features but also on the binding site conformations. All the compounds that have similar pharmacophoric features might bind to the same protein conformation, whereas the compounds with different pharmacophoric features might bind to the different conformations of the same protein. Therefore, the compounds that have similar structures and pharmacophoric features may bind to the same protein target, whereas compounds that bind to a particular protein may not have similar structures and pharmacophoric features. Hence, two compounds binding to a protein target might or might not have similar structures or pharmacophoric features.

During target enrichment, addition of targets to compounds cannot be done merely by checking whether these compounds have common targets. For instance, though bakuchicin and betulinic acid bind to DNA topoisomerase 2, it should not be simply assumed that they might have similar pharmacophoric features and thus bakuchicin may bind to the other targets of betulinic acid. This may not be true because these compounds have different pharmacophoric features. Therefore, pharmacophore analysis and docking studies are necessary and they play a key role in target enrichment.

Since we need active compounds that target multiple proteins, it would be ideal to consider the already discovered active compounds that directly target multiple proteins and then apply target enrichment methodology to further increase their protein targets. For instance, diadzein (AC42) may not be an ideal choice in anti-diabetic formulation since it targets only two proteins, namely DNA polymerase and DNA topoisomerase 2. We could check for alternate compounds that might target many other proteins apart from these two. It turns out that no other compound binds to these two targets. However, betulinic acid (AC3) that targets multiple proteins also binds to DNA topoisomerase 2, a common target for both AC3 and AC42. Therefore, by applying target enrichment studies we could check whether these compounds share similar pharmacophoric features. In case they do, we could hypothesize that since AC3 and AC42 have similar pharmacophore features and similar protein targets, AC3 may bind to DNA polymerase also. This could be verified through docking studies. If AC3 is binding to DNA polymerase, then we could enrich the targets of AC3 by adding this protein. Therefore, in the anti-diabetic formulations, instead of AC42 we could use AC3 that targets multiple proteins. This would further reduce the usage of therapeutically insignificant plants in anti-diabetic formulations.

In this study, betulinic acid, oleanolic acid, gallic acid and myricetin have high number of direct protein targets (Table S1). Therefore, target enrichment was carried out on them. For target enrichment of these compounds, we need to check for other compounds that share at least one of their protein targets.

**Figure 14:**
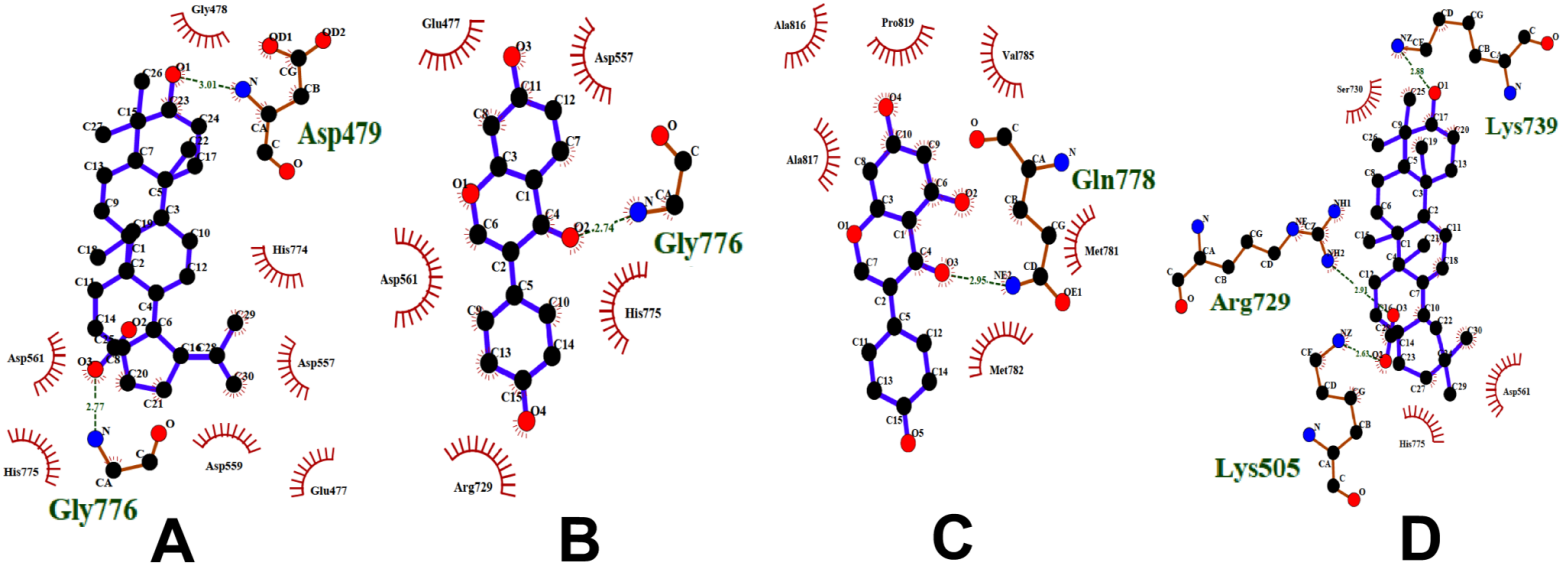
Compounds docked to DNA topoisomerase 2. (A) Betulinic acid, (B) Diadzein, (C) Genistein and (D) Oleanolic acid. Betulinic acid and diadzein bind to this protein with different features. Diadzein and genistein bind with similar features. Oleanolic acid and betulinic acid also bind with similar features.

In the case of betulinic acid, the active compound, diadzein, shares the protein target DNA topoisomerase 2 and oleanolic acid shares the protein targets, namely, glycogen phosphorylase, diacylglycerol acyltransferase and DNA polymerase beta. Betulinic acid and diadzein do not have similar pharmacophoric features. Therefore, betulinic acid might not bind to the other targets of diadzein. This was further confirmed through docking of betulinic acid and diadzein to DNA topoisomerase 2. Both these compounds bind to this protein with different features (Figure 14). During pharmacophore analysis, it was observed that among the compounds that bind to DNA topoisomerase 2, diadzein and genistein have similar pharmacophoric features. When these compounds were docked to this protein, it was found that they bind with similar features. This further supports the fact that if two compounds have similar pharmacophoric features, they bind to a common protein target, which could be proved through docking studies.

**Figure 15:**
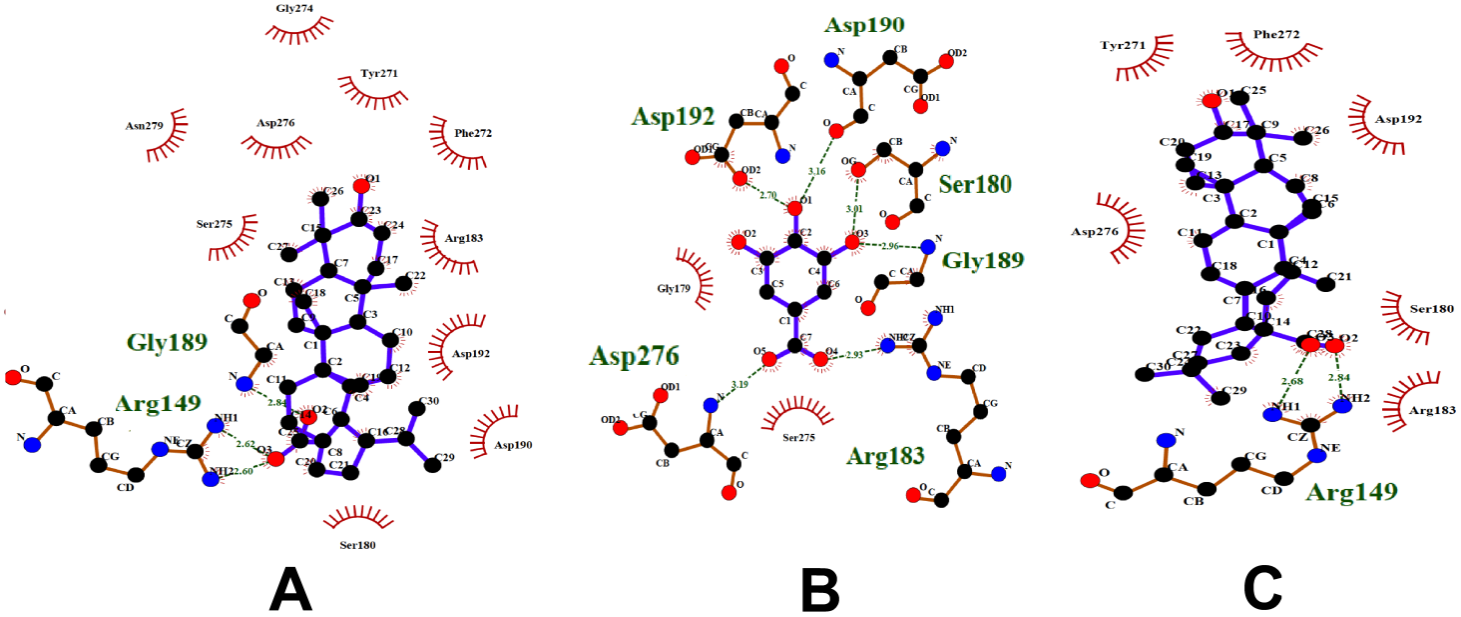
Compounds docked to DNA polymerase beta. (A) Betulinic acid, (B) Gallic acid and (C) Oleanolic acid. Betulinic acid and oleanolic bind similarly to this protein.

Betulinic acid and oleanolic acid have similar pharmacophoric features. These compounds were docked to their common target, DNA polymerase beta (Figure 15). It was observed that they bind similarly to this protein. Since compounds with similar pharmacophore features bind similarly to their protein targets and also might bind to similar protein targets as well, betulinic acid may bind to the other targets of oleanolic acid and vice versa. Thus, Gamma-Aminobutyric acid (GABA) transaminase, COX-1 and COX-2 could be added to the protein targets list of betulinic acid.

Oleanolic acid shares the targets diacylglycerol acyltransferase, glycogen phosphorylase and DNA polymerase beta with betulinic acid. It also shares COX-1 and COX-2 with gallic acid. The compounds oleanolic acid and gallic acid appear to have very few similar pharmacophoric features. Therefore, oleanolic acid might not be binding to the other proteins of gallic acid. Their mode of binding with these proteins could not be studied through docking, since these protein structures are not available. It has been observed that oleanolic acid and betulinic acid have similar pharmacophoric features and also bind similarly to DNA polymerase beta (Figure 15). So, oleanolic acid might bind to the other targets of betulinic acid. To verify this, oleanolic acid was docked to DNA topoisomerase 2. It was observed that it binds to this protein similar to betulinic acid (Figure 14). Thus, DNA topoisomerase 2, 5-alpha reductase and Liver X receptor alpha (LXR alpha) could be added to the protein targets list of oleanolic acid.

**Figure 16:**
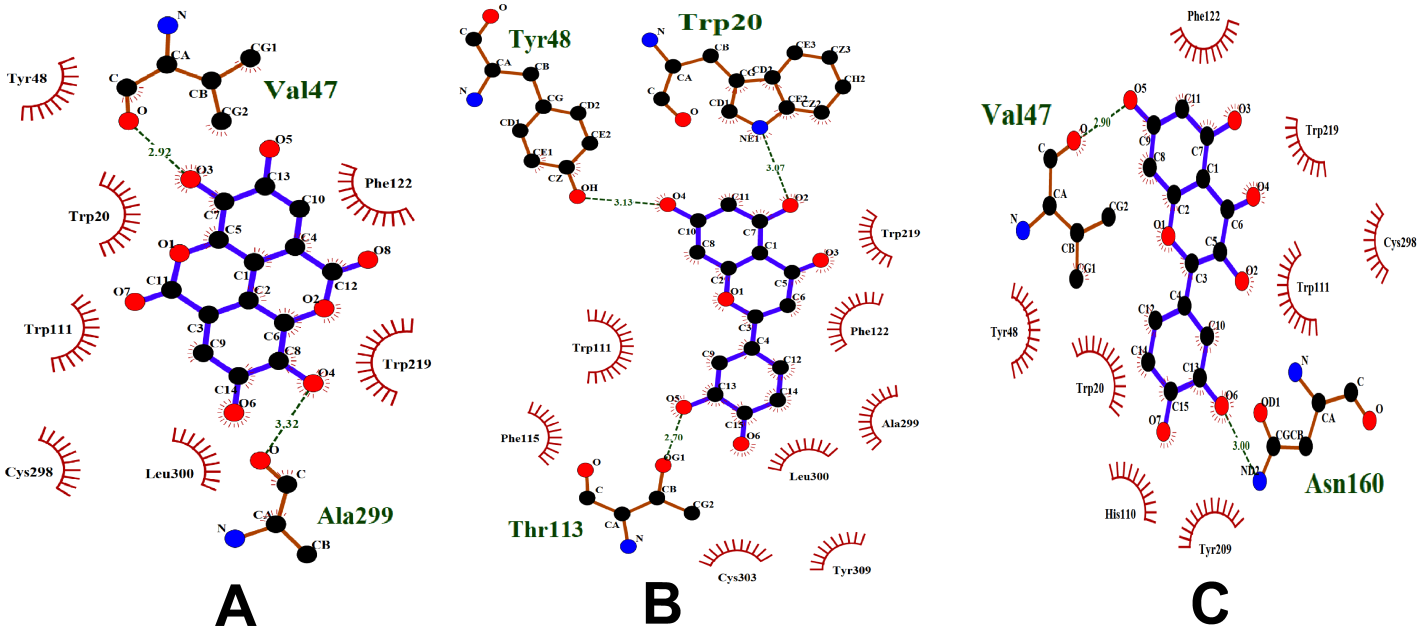
Compounds docked to aldose reductase. (A) Ellagic acid, (B) Luteolin and (C) Quercetin. All these compounds bind to this protein with similar features.

In the case of gallic acid, it shares aldose reductase with kaempferol, apigenin and luteolin. It shares COX-1 and COX-2 with oleanolic acid. During pharmacophore analysis, it was observed that all the compounds that bind to aldose reductase have similar pharmacophoric features (Figure 8). Therefore, all of them might share similar protein targets. To verify this, all these compounds were docked to aldose reductase. It was observed that they bind to it with similar features. (Figure 16). Therefore, gallic acid may bind to the other protein targets of these compounds. Thus, lipoxygenase, 20-alpha hydroxysteroid dehydrogenase and alpha glucosidase could be added to the protein targets list of gallic acid. Hence, during anti-diabetic formulations, the plants containing gallic acid could be considered instead of the plants containing kaempferol, apigenin and luteolin, since gallic acid binds to the protein targets of kaempferol, apigenin and luteolin and might exhibit effects similar to them.

**Figure 17:**
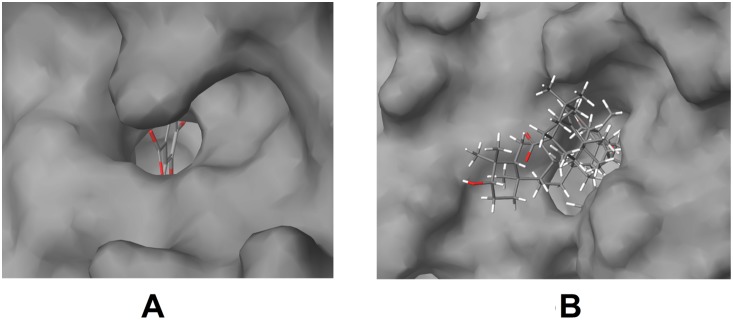
Compounds docked to aldose reductase. (A) Gallic acid, (B) Oleanolic acid and Betulinic acid. The binding pocket of aldose reductase is small. Gallic acid bound to a deep pocket whereas oleanolic acid and betulinic acid were not able to enter the pocket. Therefore, it could only accommodate gallic acid but not oleanolic acid and betulinic acid.

Though oleanolic acid and gallic acid have COX-1 and COX-2 as common targets, they do not have very similar pharmacophoric features (Figure 13). Only a part of them is similar. This shows that they might not bind to all the protein targets commonly. Oleanolic acid may bind to very few or no protein targets of gallic acid and vice versa. To study this, gallic acid was docked to DNA polymerase beta, which is a target of oleanolic acid. It was observed that gallic acid binds well to DNA polymerase beta. It binds to the amino acid residues of DNA polymerase beta, similar to oleanolic acid and betulinic acid (Figure 15). However, when oleanolic acid and betulinic acid were docked to aldose reductase, which is a target of gallic acid, they were not able to bind properly. Gallic acid bound to a deep pocket whereas oleanolic acid and betulinic acid were not able to enter the pocket of aldose reductase (Figure 17). This might be due to the fact that the binding pocket of DNA polymerase beta is large so that it could accommodate the large oleanolic acid and betulinic acid molecules as well as the small gallic acid molecule. However, the binding pocket of aldose reductase is small; therefore, it could only accommodate gallic acid but not oleanolic acid and betulinic acid (Figure 17). This indicates that when compounds are partly similar, although they share a similar protein target, they may not share all the targets of each other. Thus, target enrichment might depend on the degree of structural and feature similarity of the compounds. The higher the similarity, higher may be their protein targets being shared.

Myricetin does not share its protein targets with any other active compounds. The protein targets of myricetin are exclusive to this compound only; therefore, target enrichment could not be applied on myricetin. This should be considered one of the most important compounds.

### 8. Predicted ADME properties

The ADME and pharmacokinetic properties of compounds are very important. We analyzed physically significant descriptors and pharmaceutically relevant properties of all the active compounds (Table S2). The number of stars, which represent the number of property or descriptor values that fall outside the 95% range of similar values for known drugs, were within the recommended range of 0–5 for almost all the compounds. Molecules with a large number of stars suggest that they are less drug-like than those with few stars. The molecular weight of all the compounds is within the recommended range of 130–725. The octanol/water partition coefficient (QP logPo/w) and aqueous solubility (QP logS), which are essential in the estimation of absorption and distribution of drugs within the body, ranged between −5.814 to 7.396 and −8.947 to 0.306, respectively. Most of the compounds were within the acceptable range of −2.0–6.5 for QP logPo/w and −6.5–0.5 for QP logS. The key factors, namely QPPCaco, which governs the drug metabolism and its access to biological membranes, and QPPMDCK, which predicts the cell permeability of the drugs, were within the recommended range for most of the compounds (values <25 are poor and >500 are good). The % human oral absorption for most of the compounds was within the range of 25–80 (<25% is poor and >80% is high). Further, all the compounds were violating Lipinski’s rule of five in the range of 0–2 (maximum number of violations acceptable are 4). Thus, most of the pharmacokinetic parameters of the compounds are within the acceptable range for human use.

## Discussion

Through network analysis, we have attempted to show the biological means of pharmacological action of anti-diabetic plants. It was observed that most of the active compounds present in anti-diabetic plants act on multiple protein targets. These proteins in turn are involved in multiple pathways related to diabetes and their complications. Network analysis revealed important information like 1) the anti-diabetic plants that contain many active compounds, 2) the active compounds that are present in most of the anti-diabetic plants, 3) the active compounds that target most of the proteins, 4) proteins that are targeted by most of the active compounds, 5) proteins that are involved in multiple pathways, 6) pathways that involve most of the proteins, 7) proteins that are involved in different diabetes-related complications and 8) the proteins that are involved in various other diseases. It was observed that these diseases share similar pathophysiological mechanisms with diabetes. This study provided an overview of the proteins and the pathways involved in diabetes. Thus, the mechanism of action of anti-diabetic plants and their active compounds was deduced by analyzing their protein targets and respective pathways. For instance, gallic acid was reported to have anti-lipid peroxidative and anti-lipidemic effects in diabeteic rats [29], [30]. In this study, we have shown that gallic acid binds to protein targets PT16, PT23, PT24, PT25 and PT26. The protein PT16 is involved in glycerolipid metabolism pathway, P6. The proteins PT24 and PT25 are involved in the metabolism of a fatty acid namely arachidonic acid. The involvement of protein targets in lipid metabolism pathways explains the role of gallic acid in anti-lipid peroxidative and anti-lipidemic effects.

**Figure 18:**
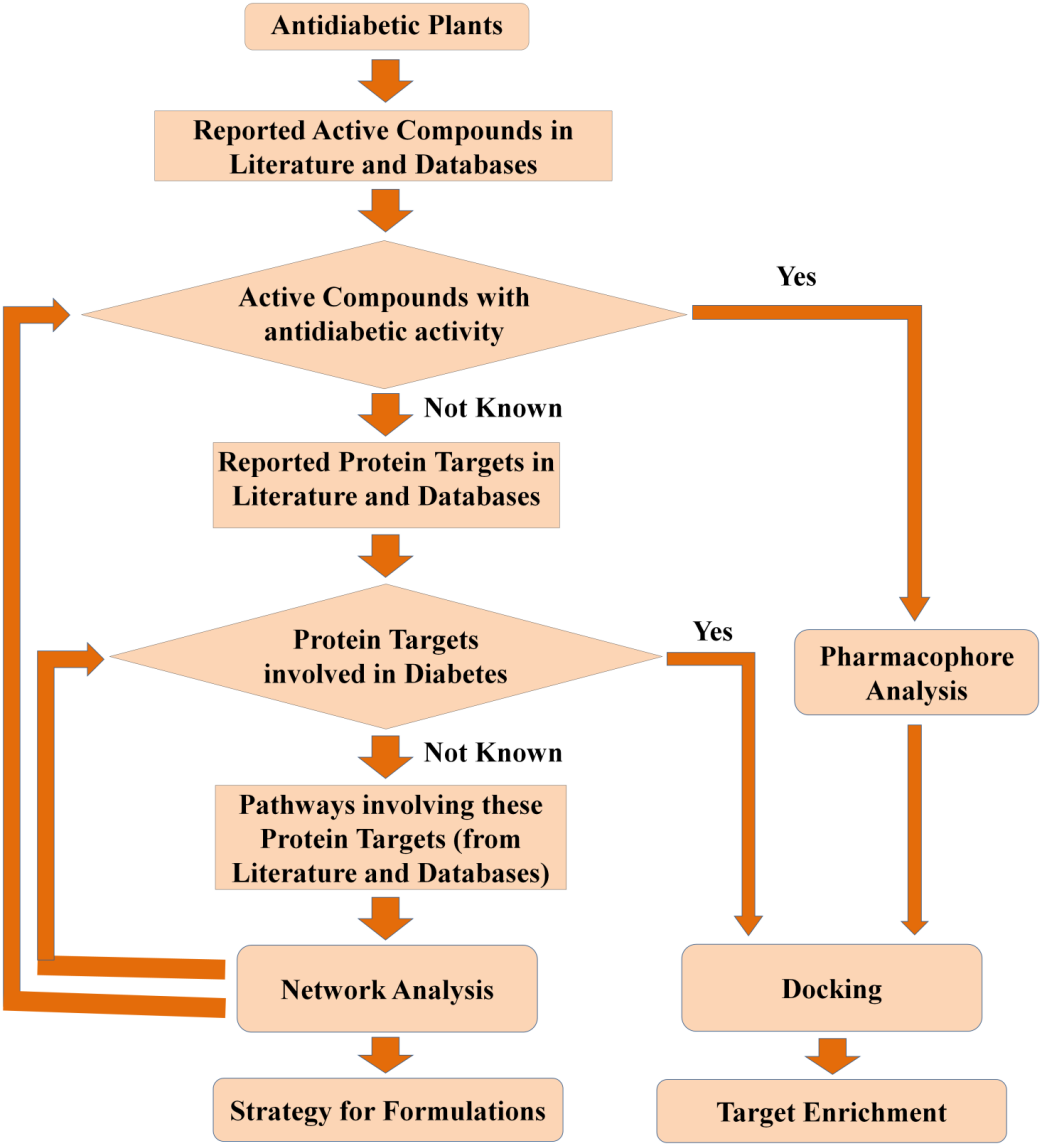
The step-by-step methodology used in this study. The active compounds of anti-diabetic plants, their protein targets, and the pathways in which these proteins are involved were retrieved from literature and databases. If the anti-diabetic activity of the active compounds is not reported/known, then it is identified through network analysis by analyzing whether their protein targets and respective pathways are involved in diabetes. The target enrichment of active compounds with anti-diabetic activity was carried out through pharmacophore analysis and docking. If the involvement of protein targets in diabetes is not known, then it is identified through network analysis by analyzing whether their respective pathways are involved in diabetes. The protein targets involved in diabetes were employed in docking for target enrichment.

Pharmacophore analysis has revealed the chemical basis of pharmacological action of anti-diabetic plants. It was observed that the anti-diabetic plants that show similar mechanisms of action contain active compounds with similar pharmacophoric features. These active compounds in turn bind to similar protein targets and thus show similar pharmacological action. Further, through pharmacophore analysis and docking studies, we have introduced a target enrichment protocol to discover novel protein targets for the important active compounds. The step-by-step methodology used in this study is represented in the form of a flow chart, which also reflects the decision making at various stages (Figure 18).

In this study we have proposed various hypotheses that have been derived from network analysis. Observations made from them revealed that the active compounds that target multiple proteins and also that target proteins which are involved in multiple pathways are important for effective anti-diabetic activity. Taking this in to consideration, we have proposed a strategy that could be used in the preparation of new effective anti-diabetic formulations.

From this study, it could be deduced that an anti-diabetic formulation containing active compounds AC2, AC3, AC5, AC6, AC11, AC54 and AC58, which are present in the anti-diabetic plants ADP2, ADP3, ADP5, ADP23 and ADP25, might have better anti-diabetic activity. The significance of these compounds and their possible mechanism of action against diabetes are as follows.

### AC2 (1-deoxynojirimycin)

AC2 was reported as an alpha-glucosidase inhibitor, but little or no information is available regarding its anti-diabetic activity. It is structurally similar to known FDA approved alpha-glucosidase inhibitors, Miglustat and Miglitol (Figure S1). Miglustat (N-butyl-deoxynojirimycin) is used primarily to treat Type-1 Gaucher disease (GD1). It is an imino sugar, a synthetic analogue of D-glucose. Miglitol is an oral anti-diabetic drug that acts by inhibiting the ability of the patient to break down complex carbohydrates into glucose. It is primarily used in T2D for establishing greater glycemic control by preventing the digestion of carbohydrates (such as disaccharides, oligosaccharides, and polysaccharides) into monosaccharides, which can be absorbed by the body. Miglustat and Miglitol inhibits alpha-glucosidases. Since AC2 also inhibits alpha-glucosidases, its mechanism of action might be similar to these drugs.

AC2 targets alpha glucosidases directly. Alpha glucosidase is a carbohydrate-hydrolase that breaks down starch and disaccharides to glucose. It is involved in N-glycan biosynthesis (P3) and sucrose metabolism (P20) pathways. Alpha glucosidases are very well known enzymes involved in diabetes and their inhibitors were reported to show good anti-diabetic effects. Alpha glucosidase inhibitors could inhibit the absorption of carbohydrates from the gut and are used in the treatment of patients with T2D or impaired glucose tolerance. It was reported that inhibition of intestinal alpha-glucosidases reduced the rate of glucose absorption by delaying the carbohydrate digestion and prolonging the overall carbohydrate digestion time [31]. Further, the postprandial rise in blood glucose was dose-dependently decreased, and glucose-induced insulin secretion was also attenuated. Thus, alpha glucosidase inhibition was proved to have potential in delaying and possibly preventing the development of diabetic complications [31]. AC2 was reported to strongly inhibit alpha glucosidases [32]. Therefore, inhibition of alpha glucosidases might be an important reason behind the anti-diabetic activity of AC2.

AC2 targets glycogen debranching enzyme, cytosolic beta glucosidase, lactase-glycosylceramidase and glucosidase II beta subunit precursor enzyme indirectly through glucosidases. Glycogen debranching enzyme is involved in glycogenolysis. Conversion of glycogen to glucose-1-phosphate occurs in glycogenolysis that leads to the increase in blood glucose levels. The role of cytosolic beta glucosidase in diabetes is not reported in the literature. But, from our data analysis, it can be seen that cytosolic beta glucosidase is involved in starch and sucrose metabolism, which is an important pathway related to diabetes. Similarly, the role of glycosylceramidase in diabetes is also not known. The substrates of this enzyme are glycosyl-N-acylsphingosine and H_2_O, whereas its two products are N-acylsphingosine and sugar. Further, it is involved in galactose metabolism pathway. Therefore, glycosylceramidase may lead to an increase in sugar levels, which is targeted by AC2.

### AC3 (Betulinic acid)

AC3 is a naturally occurring pentacyclic triterpenoid, having anti-retroviral, anti-malarial and anti-inflammatory properties. Recently, it has been discovered as a potential anti-cancer agent [33]. Since it functions as both a hypolipidemic and a hypoglycemic agent, it was proposed that AC3 may have therapeutic potential in combating T2D and obesity, by effectively modulating the various enzymes and hormones involved in the absorption and metabolism of carbohydrates and lipids [34]. Betulinic acid and oleanolic acid, both pentacyclic triterpenoids, have shown multiple biological activities with apparent effects on glucose absorption, glucose uptake, insulin secretion, diabetic vascular dysfunction, retinopathy and nephropathy. The versatility of the pentacyclic triterpenes provides a promising approach for diabetes management [35].

AC3 directly targets six proteins: diacylglycerol acyltransferase, 5-alpha reductase, glycogen phosphorylase, DNA topoisomerase 2, DNA polymerase beta and LXR alpha. The first enzyme, diacylglycerol acyltransferase, catalyzes the final step in triglyceride synthesis. Pharmacological studies suggest that inhibition of this enzyme is a promising strategy for the treatment of T2D [36]. It was reported that the mice with hepatic overexpression of diacylglycerol acyltransferase showed hepatic insulin resistance [36]. This enzyme is also involved in glycerolipid metabolism. The second enzyme, 5-alpha reductase, is involved in steroid metabolism. It converts testosterone to 5α-dihydrotestosterone (DHT) in peripheral tissues. The role of this enzyme in diabetes is not well known. It is involved in steroid hormone biosynthesis pathway. The natural mechanism of 5-alpha reductase inhibition involves the binding of nicotinamide adenine dinucleotide phosphate (NADPH) to the enzyme followed by the substrate. The structure of AC3 is similar to the known synthetic drugs of 5-alpha reductase, namely, finasteride and dutasteride (Figure S2). These drugs are mainly used for the treatment of benign prostatic hyperplasia (BPH) and male pattern baldness (MPB).

The third enzyme targeted by AC3, glycogen phosphorylase, catalyzes the rate-limiting step in glycogenolysis. It breaks up glycogen into glucose subunits. It was reported that inhibition of hepatic glycogen phosphorylase is a promising treatment strategy for attenuating hyperglycemia in T2D [37]. The regulation of the hepatic glucose output through glycogenolysis is an important target for T2D therapy. This mechanism of action of AC3 is similar to AC2, which also targets glycogenolysis through glycogen debranching enzyme. The fourth enzyme, DNA topoisomerase 2, cut the strands of the DNA helix in order to manage DNA tangles and supercoils. It was reported that chronically elevated levels of glucose increases the mitochondrial DNA damage and thus contributes to mitochondrial dysfunction [38]. Mitochondria-dependent DNA cleavage was significantly exacerbated and mitochondrial topoisomerase function was significantly altered in the presence of hydrogen peroxide (H_2_O_2_). Further, when mitochondria were chronically exposed to elevated glucose, significant increase in topoisomerase-linked DNA cleavage was observed [38]. Therefore, inhibition of this topoisomerase by compounds like AC3 might have beneficial effects in the case of diabetes. DNA polymerase beta is another enzyme involved in the maintenance of DNA, during DNA damage, through base excision repair. The sixth protein, LXR alpha, is a sensor of cholesterol metabolism and lipid biosynthesis. It is an important regulator of cholesterol, triglyceride and glucose homeostasis. It has been reported that LXR is a highly interesting target for drug development for treating diabetes [39].

AC3 indirectly targets nitric oxide synthase (NOS) and NADPH oxidase. Impaired NOS pathway was seen in diabetes mellitus. It was reported that endothelial nitric oxide synthase (eNOS) deficiency produced accelerated nephropathy in diabetic mice [40]. Similarly, eNOS knockout in diabetic mice developed advanced diabetic nephropathy [41]. Therefore, a higher level of NOS is desirable to reduce diabetic complications. AC3 was reported to up-regulate eNOS. AC3-treated endothelial cells showed an increased production of bioactive nitric oxide [42]. NADPH oxidase is an important protein involved in diabetes. It catalyzes the conversion of NADPH to NADP, thereby producing superoxide free radicals. These free radicals in turn are involved in DNA damage and in the impairment of the function of DNA repairing enzymes such as DNA topoisomerase 2 and DNA polymerase beta. AC3 downregulates the NADPH oxidase levels [42]. As it was mentioned previously that the natural mechanism of inhibition of 5-alpha reductase involves the binding of NADPH to that enzyme, presence of NADPH oxidase may lower the concentration of NADPH and thus decreases 5α reductase inhibition. However, inhibition of NADPH oxidase not only reduces the formation of free radicals but also increases the levels of NADPH. Therefore, AC3 may inhibit 5α reductase both directly through binding to it and indirectly by inhibiting NADPH oxidase.

### AC5 (Oleanolic acid)

AC5 is a naturally occurring triterpenoid, widely distributed in food and medicinal plants. It is relatively non-toxic, hepatoprotective, and exhibits anti-tumor and anti-viral properties [43]. AC5 directly targets diacylglycerol acyltransferase, glycogen phosphorylase, GABA transaminase, cyclooxygenase-1 (COX-1), cyclooxygenase-2 (COX-2) and DNA polymerase beta. The proteins diacylglycerol acyltransferase, glycogen phosphorylase and DNA polymerase beta have been discussed with respect to AC3 since they share common targets. Thus, AC5, by targeting these proteins, may show mechanism of action similar to AC3. Among the remaining proteins, GABA transaminase is exclusively targeted by AC5. This enzyme is involved in amino acid metabolism pathways and catalyzes the conversion of 4-aminobutanoate to amino acids such as alanine, glycine and glutamate. It was reported that alterations in the alanine cycle and increase in the levels of serum alanine aminotransferase (ALT) is linked to the development of T2D. Further, elevated level of ALT increases the risk of developing T2D [44]. Inhibition of GABA transaminase would in turn inhibit alanine formation. This enzyme also forms glutamate whose abnormal homeostasis is commonly observed in diabetes. Abnormal glutamate homeostasis might contribute to diabetes pathogenesis and excessive glutamate consumption might cause insulin resistance [45]. Further, high extracellular glutamate levels exert direct and indirect effects that might participate in the progressive loss of beta-cells occurring in both T1D and T2D [45]. Therefore, inhibition of GABA transaminase by AC5 might reduce the formation of glutamate and its diabetic complications.

Glycine is formed from the reaction between 4-aminobutanoate and glyoxylate, catalyzed by GABA transaminase. Theoretically, inhibition of GABA transaminase would inhibit this reaction and thereby reduces the formation of glycine. Interestingly, glycine shows anti-diabetic effects. Patients with uncontrolled T2D have severely deficient synthesis of glutathione (GSH). Dietary supplementation with glycine restored GSH synthesis and lowered oxidative stress and oxidant damage in the face of persistent hyperglycemia [46]. Further, glycine reduced the alterations induced by hyperglycemia in streptozotocin-induced diabetic rats [47]. Moreover, glyoxylate, one of the substrates in the above mentioned GABA transaminase catalyzed reaction was reported to be at significantly higher levels in diabetes patients. It was proposed that glyoxylate could be used as a potential novel marker for early detection of T2D [48]. Therefore, a higher level of GABA transaminase is needed to produce glycine through glyoxylate-based reaction. Hence, inhibition of this enzyme by AC5 is not desirable. However, the relation between GABA transaminase, glyoxylate and glycine has not been practically studied. Since the role of GABA transaminase in diabetes is not clearly known, this would be an interesting study to understand the mechanism of action of this enzyme. The enzymes COX-1 and COX-2 are responsible for the conversion of arachidonic acid to prostanoids. COX-1 is the rate-limiting enzyme in the control of prostanoid metabolism. Altered prostanoid metabolism is seen in the pathogenesis of diabetic complications. Therefore, targeting of COX-1 and COX-2 enzymes might be one of the reasons behind the anti-diabetic activity of AC5. These enzymes are involved in arachidonic acid metabolism pathway. It was reported that inhibitors of these enzymes would have a dual protective role in diabetes, by minimizing beta-cell dysfunction and by maintaining insulin secretion through enhancing endogenous arachidonic acid levels [49].

### AC6 (Myricetin)

AC6 is a natural bioflavonoid whose occurrence in nature is widespread among plants. It has been demonstrated to possess both antioxidative and prooxidative properties. It is a potent anti-carcinogen and anti-mutagen. It has therapeutic potential and benefits in cardiovascular diseases and diabetes mellitus [50]. AC6 provided protection against oxidative stress in T2D erythrocytes [51]. It had beneficial effect on renal functions in streptozotocin-induced diabetes and thus, it was suggested that AC6 could be of therapeutic potential in diabetic nephropathy [52].

AC6 targets alpha-amylase, xanthine oxidase, insulin receptor and phosphoinositide 3-kinase (PI3K). These proteins are exclusively targeted by this active compound. Alpha-amylase plays an important role in the digestion of starch and glycogen. This enzyme is involved in pancreatic secretion, starch and sucrose metabolism pathways. One of the therapeutic strategies for the treatment of T2D includes the inhibition of degradation of oligo and disaccharides. Phytochemicals as inhibitors of alpha-amylase has been proposed as a possibility to treat diabetes mellitus [53]. Further, significant reduction in the post-prandial increase of blood glucose was proposed as an important strategy in the management of blood glucose level in T2D [54]. Reactive oxygen species (ROS), such as superoxide, hydroxyl radical, and hydrogen peroxide arise from many cellular sources in response to hyperglycemia and diabetes. These sources include oxidative phosphorylation, glucose autoxidation, NADPH oxidase, and other enzymes such as xanthine oxidase. The role of NADPH oxidase in diabetes has been described in the earlier paragraphs and it was also mentioned that one of the reasons behind the anti-diabetic activity of AC3 is the down regulation of NADPH oxidase levels. Xanthine oxidase catalyzes the oxidation of hypoxanthine to xanthine and further catalyzes the oxidation of xanthine to uric acid. Superoxide, hydrogen peroxide, and hydroxyl radicals are produced as byproducts of the xanthine oxidase reaction. Treatment with allopurinol, a xanthine oxidase inhibitor, decreased oxidative stress in T1D patients [55]. Inhibition of xanthine oxidase was reported to attenuate the development of diabetic cardiomyopathy [56]. Further, inhibition of xanthine oxidase reduced hyperglycemia-induced oxidative stress and improved mitochondrial alterations in skeletal muscle of diabetic mice [57]. Therefore, inhibition of these proteins might attribute anti-diabetic effect to AC6.

Another enzyme that is targeted by AC6 is the insulin receptor. The insulin receptor (IR) is activated by insulin and plays a key role in the regulation of glucose homeostasis. Activation of the insulin receptor induces glucose uptake. A decrease in insulin receptor signaling or insulin insensitivity leads to T2D. In this case, the cells would be unable to take up glucose and would result in hyperglycemia. AC6 was found to ameliorate defective post-receptor insulin signaling through the enhancement of β-endorphin secretion [58]. AC6 attenuated hyperinsulinemia-induced insulin resistance in skeletal muscle cells. It also ameliorated insulin resistance induced by a high-fructose diet in rats. AC6 improved insulin sensitivity through the enhancement of insulin action on insulin receptor substrate-1 (IRS-1) associated phosphoinositide 3-kinase (PI3-kinase) and glucose transporter type 4 (GLUT4) activities in the muscles of animals exhibiting insulin resistance [59].

### AC11 (Gallic acid)

AC11 exhibited anti-hyperglycemic, anti-lipid peroxidative, antioxidant, and anti-lipidemic effects in streptozotocin-induced type 2 diabetic rats [60]. It could provide a beneficial effect on diabetes by decreasing oxidative stress-related diabetic complications [61]. It was proposed that a diet containing gallic acid might be beneficial to T2D patients [60]. Cardioprotective effects of AC11 were observed in diabetes-induced myocardial dysfunction in rats. Thus, it was suggested that AC11 could be beneficial for the treatment of myocardial damage associated with T1D [62]. AC11 targets aldose reductase, UDP glucose dehydrogenase, ribonucleotide reductase, COX-1 and COX-2. Aldose reductase (AR) is widely expressed aldehyde-metabolizing enzyme. Aldose reductase catalyzes the reduction of a variety of aldehydes and carbonyls. It primarily catalyzes the reduction of glucose to sorbitol, the first step in polyol pathway of glucose metabolism. The reduction of glucose by the AR-catalyzed polyol pathway has been linked to the development of secondary diabetic complications. Glucose concentrations are often elevated in diabetics and AR was believed to be responsible for many diabetic complications. AR is involved in fructose, mannose, galactose and glycerolipid metabolism pathways. A study on the role of AR and the polyol pathway in diabetic nephropathy in a transgenic rat model showed that AR may both exacerbate and alleviate the production of metabolites that lead to hyperglycemia-induced cellular impairment [63]. Elevated level of AR expression was induced by hyperglycemia in patients with diabetic nephropathy [64]. Fidarestat, an AR inhibitor prevented diabetes-associated cataract formation, retinal oxidative-nitrosative stress, glial activation and apoptosis in streptozotocin induced diabetic rats [65]. AC11 was found to inhibit AR effectively [66] and this might be one of the reasons behind the effective anti-diabetic property of the plants containing this compound.

UDP glucose dehydrogenase is involved in starch and sucrose metabolism pathways. 40–50% increase in the expression of this enzyme was observed in streptozotocin-induced diabetic rats [67]. AC11 was reported to be an effective inhibitor of UDP glucose dehydrogenase [68]. Ribonucleotide reductase (RNR) catalyzes the formation of deoxyribonucleotides from ribonucleotides. RNR plays a critical role in DNA synthesis and repair. A high frequency of mitochondrial DNA (mtDNA) mutations is seen in T2D. During high DNA damage, similar to DNA topoisomerase 2 and DNA polymerase beta, the function of RNR gets impaired and leads to diabetic complications [69]. AC11 could significantly inhibit RNR [70]. The role of COX-1 and COX-2 in diabetes has been discussed previously. It was reported that AC11 could effectively inhibit COX-1 and COX-2 enzymes [70]. Taken together, AC11 might be exhibiting anti-diabetic activity by targeting these proteins.

### AC54 (Shogaol)

AC54 is a pungent constituent of ginger (*Zingiber officinale*), similar in chemical structure to gingerol. AC54 targets nuclear factor kappa B (NF-kB), COX-2 and mitogen-activated protein kinase (MAPK). NF-kB mainly controls the transcription of DNA. A study involving NF-kB activation and overexpression of its regulated genes in patients with T2D revealed that the activation of NF-kB and the transcription of certain pro-inflammatory chemokines in tubular epithelial cells are markers of progressive diabetic nephropathy [71]. Apoptotic beta-cell death is the basic reason behind the pathogenesis of T1D and also in the islet graft rejection. It was reported that cell-death might be mediated by the activation of the NF-kB pathway. Use of NF-kB inhibitor significantly reduced apoptosis [72]. Beta-cell-specific activation of NF-kB was found to be a key event in the progressive loss of beta-cells in diabetes. Inhibition of this process was suggested as a potential effective strategy for beta-cell protection [72]. Administration of the antioxidants to the alloxan diabetic rats repressed the activation of NF-kB. *In vitro* studies showed that high glucose activates NF-kB and elevates NO and lipid peroxides in both retinal endothelial cells and pericytes. Inhibition of NF-kB and its downstream pathways had beneficial effects on the development of diabetic retinopathy [73]. Therefore, inhibition of NF-kB by AC54 might impart it anti-diabetic activity.

MAPK is another protein targeted by AC54. It is an important mediator of the extracellular stimuli, implicated in the pathogenesis of early diabetes mellitus. Elevated MAPK activity was observed in streptozotocin-induced diabetes mellitus rats [74]. The proteins p38 kinase (p38) and c-Jun NH2-terminal kinase (JNK) belong to the family of MAPK and are activated in response to hyperglycemia, oxidative stress, and proinflammtory cytokines. Increased activation of p38 and JNK have been reported as a fundamental mechanism responsible for cardiovascular dysfunction in diabetes [75], [76]. Inhibition of p38/JNK improved nitric oxide–mediated vasodilatation and reduced inflammation in hypercholesterolemic patients [77]. MAPKs were implicated in the etiology of diabetic neuropathy both *via* direct effects of glucose and indirectly through glucose-induced oxidative stress [78]. It was reported that p38 MAPK inhibition reduced diabetes-induced impairment of wound healing. This was achieved by p38 MAPK inhibitors which demonstrated anti-inflammatory effects, primarily by inhibiting the expression of inflammatory cytokines and regulating cellular traffic into the wounds. It was suggested that chronic wounds associated with diabetes could be managed by means of p38 MAPK inhibitors [79]. p38 MAPK also plays an important role in diabetes-induced inflammation in the retina. Streptozotocin-induced insulin-deficient diabetic control rats exhibited significant increases in retinal superoxide, nitric oxide (NO), COX-2, and leukostasis within retinal microvessels. All these abnormalities were significantly inhibited by a p38 MAPK inhibitor [80]. It was suggested that inhibition of p38 MAPK would offer a novel therapeutic approach to inhibiting the development of early stages of diabetic retinopathy and other complications of diabetes. AC54 significantly inhibited the function of NF-kB and also the expression of COX-2 and MAPK [81]. The role of COX-2 in diabetes has been discussed previously. Therefore, the anti-diabetic activity of AC54 might be due to its inhibition of NF-kB, COX-2 and MAPK, and this might have attributed the anti-diabetic property to the plants containing this compound.

### AC58 (Wedelolactone)

AC58 targets alpha glucosidase and inhibitor of NF-kB kinase subunit beta (IKK-beta). AC58 inhibited adipogenesis through the IKK-beta and the ERK pathway in human adipose tissue-derived mesenchymal stem cells [82]. The metabolic disorders of insulin resistance and T2D are mainly caused due to inflammation. IKK-beta was found to be a central coordinator of inflammatory response through the activation of NF-kB. IKK-beta acted locally on liver and systemically on myeloid cells, where NF-kB activation induced inflammatory mediators caused insulin resistance in mice [83]. Therefore, IKK-beta was thought to link inflammation to obesity-induced insulin resistance. It was suggested that inhibition of IKK-beta might be used to treat insulin resistance [83]. The role of alpha glucosidase in diabetes has been discussed previously at AC2. One of the effective therapeutic approaches in the treatment of diabetes mellitus is decreasing the postprandial hyperglycemia through the inhibition of alpha glucosidase. AC58 exhibited intensive inhibitory effect on alpha glucosidase [84]. Oral administration of AC58 could significantly decrease blood glucose after maltose loading in the diabetic rats in a dose-dependent manner. Thus, it was proposed that AC58 might reduce the postprandial glucose level and exert an anti-diabetic effect by suppressing carbohydrate absorption from the intestine [84]. Thus, inhibition of alpha glucosidase and IKK-beta might be the reason behind the anti-diabetic activity of AC58 and also for the anti-diabetic property of the plants containing AC58.

Network analysis and pharmacophore-based studies would help us in understanding the biological and chemical basis of pharmacological action of anti-diabetic plants. Further, this study also provides the framework for synthetic modification of bioactive phytochemicals, as well as delineates possible modifications of the active compounds to increase potency or selectivity, since some of the active compounds have been already structurally modified to produce effective synthetic drugs (Figures S1 and S2). Thus, an integrated approach of network analysis combined with pharmacophore analysis and docking studies could be used to discover potential active compounds. The combined use of these compounds either in the form of plant formulations or pharmaceutical drugs might show synergistic effect against multiple mechanisms involved in diabetes and its complications.

## Supporting Information

Figure S1 **Alpha-glucosidase inhibitors.** (A) AC2 (1-deoxynojirimycin), (B) Miglustat and (C) Miglitol. The synthetic drugs, miglustat and miglitol, are structurally similar to and derived from the anti-diabetic plant compound AC2.
(TIF)

Figure S2 **5α reductase inhibitors.** (A) Betulinic acid, (B) Finasteride and (C) Dutasteride. The synthetic drugs, finasteride and dutasteride, are structurally similar to and derived from the anti-diabetic plant compound betulinic acid.
(TIF)

Table S1 **Active compounds that directly target multiple proteins.**
(DOC)

Table S2 **ADME properties of active compounds.**
(DOC)
