# Supplementary figures and images for: Finding Needles in a Haystack: Application of Network Analysis and Target Enrichment Studies for the Identification of Potential Anti-Diabetic Phytochemicals
Source: PLoS One. 2014 Nov 14;9(11):e112911. doi: 10.1371/journal.pone.0112911 (PMC4232558; doi:10.1371/journal.pone.0112911)

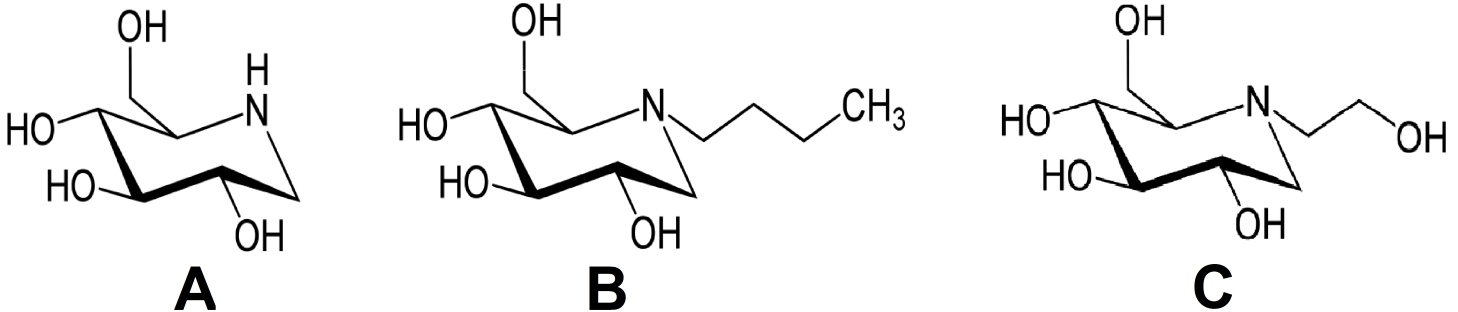

Supplement: Figure S1 — Alpha-glucosidase inhibitors. (A) AC2 (1-deoxynojirimycin), (B) Miglustat and (C) Miglitol. The synthetic drugs, miglustat and miglitol, are structurally similar to and derived from the anti-diabetic plant compound AC2. (TIF) [file pone.0112911.s001.tif]

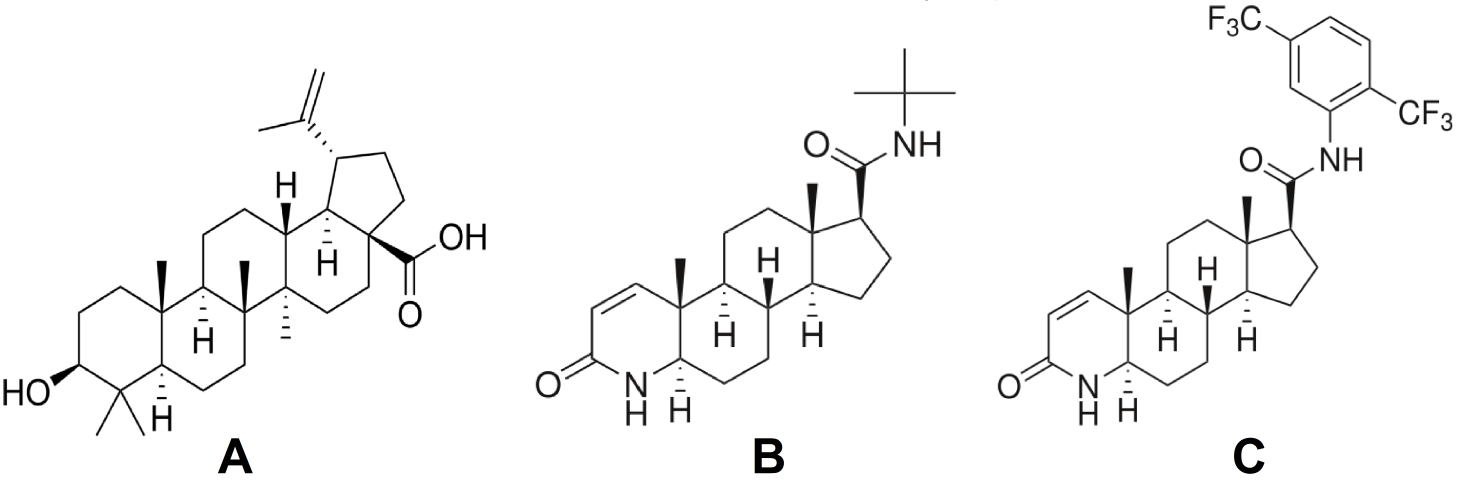

Supplement: Figure S2 — 5α reductase inhibitors. (A) Betulinic acid, (B) Finasteride and (C) Dutasteride. The synthetic drugs, finasteride and dutasteride, are structurally similar to and derived from the anti-diabetic plant compound betulinic acid. (TIF) [file pone.0112911.s002.tif]
