# Supplementary material for: Finding Needles in a Haystack: Application of Network Analysis and Target Enrichment Studies for the Identification of Potential Anti-Diabetic Phytochemicals
Source: PLoS One. 2014 Nov 14;9(11):e112911. doi: 10.1371/journal.pone.0112911 (PMC4232558; doi:10.1371/journal.pone.0112911)
Supplement: Table S1 — Active compounds that directly target multiple proteins. (DOC) [file pone.0112911.s003.doc]

| **Active Compound** | **Protein Target** |
| --- | --- |
| Betulinic acid | Glycogen phosphorylase |
|  | DNA topoisomerase 2 |
|  | 5-Alpha reductase |
|  | Diacylglycerol acyltransferase |
|  | DNA polymerase beta |
|  | LXR alpha |
| Oleanolic acid | Gaba transaminase |
|  | Diacylglycerol acyltransferase |
|  | Glycogen phosphorylase |
|  | DNA polymerase beta |
|  | COX-1 |
|  | COX-2 |
| Gallic acid | Aldose reductase |
|  | UDP glucose dehydrogenase |
|  | Ribonucleotide reductase |
|  | COX-1 |
|  | COX-2 |
| Myricetin | ALPHA AMYLASE |
|  | XANTHINE OXIDASE |
|  | INSULIN RECEPTOR |
|  | PI3K |
