# Supplementary material for: Finding Needles in a Haystack: Application of Network Analysis and Target Enrichment Studies for the Identification of Potential Anti-Diabetic Phytochemicals
Source: PLoS One. 2014 Nov 14;9(11):e112911. doi: 10.1371/journal.pone.0112911 (PMC4232558; doi:10.1371/journal.pone.0112911)
Supplement: Table S2 — ADME properties of active compounds. (DOC) [file pone.0112911.s004.doc]

| **Active Compound** | **Pubchem ID** | **Stars** | **Molecular weight** | **QP log (Po/w)** | **QP logS** | **QPP**  **Caco** | **QPP**  **MDCK** | **% Human oral absorption** | **Lipinski’s rule**  **of five** |
| --- | --- | --- | --- | --- | --- | --- | --- | --- | --- |
| Protocatechuic acid | 72 | 0 | 154.122 | 0.014 | -0.779 | 27.436 | 12.906 | 52.771 | 0 |
| Gallic acid | 370 | 0 | 170.121 | -0.585 | -0.681 | 10.027 | 4.348 | 41.441 | 0 |
| Guaiacol | 460 | 4 | 124.139 | 1.229 | -0.881 | 3005.194 | 1625.076 | 96.388 | 0 |
| Pipecolic acid | 849 | 3 | 129.158 | -1.807 | -0.525 | 46.999 | 25.548 | 46.291 | 0 |
| Palmitic Acid | 985 | 3 | 256.428 | 5.303 | -5.64 | 231.932 | 129.663 | 87.371 | 1 |
| Methoxsalen | 4114 | 0 | 216.193 | 1.561 | -1.657 | 2051.793 | 1075.795 | 95.365 | 0 |
| Stearic acid | 5281 | 3 | 284.481 | 4.674 | -8.947 | 1237.769 | 622.992 | 100 | 0 |
| Estradiol | 5757 | 0 | 272.386 | 4.008 | -4.684 | 1248.35 | 628.75 | 100 | 0 |
| Folic Acid | 6037 | 5 | 441.402 | -0.42 | -3.817 | 0.033 | 0.012 | 0 | 2 |
| Synephrine | 7172 | 0 | 167.207 | 0.198 | -0.199 | 193.322 | 92.637 | 69.025 | 0 |
| Vanillic Acid | 8468 | 0 | 168.149 | 1.058 | -1.274 | 93.613 | 48.632 | 68.424 | 0 |
| Oleanolic Acid | 10494 | 2 | 456.707 | 6.18 | -6.845 | 333.602 | 192.068 | 95.334 | 1 |
| Angelicin | 10658 | 0 | 186.167 | 0.963 | -1.267 | 2091.058 | 1098.065 | 92.01 | 0 |
| 1-deoxynojirimycin | 29435 | 1 | 219.28 | -1.169 | -0.025 | 63.393 | 27.757 | 52.355 | 0 |
| Betulinic acid | 64971 | 2 | 456.707 | 6.2 | -6.712 | 342.203 | 197.425 | 95.647 | 1 |
| Allicin | 65036 | 2 | 162.264 | 1.354 | 0.306 | 169.141 | 3941.564 | 74.754 | 0 |
| Gallocatechin | 65084 | 0 | 306.271 | -0.233 | -2.382 | 18.691 | 6.702 | 35.382 | 1 |
| Delphinidin | 68245 | 1 | 338.701 | -0.582 | -0.848 | 59.362 | 31.69 | 42.32 | 1 |
| Catechin | 72276 | 1 | 290.272 | 0.454 | -2.518 | 58.241 | 22.893 | 61.197 | 0 |
| Epigallocatechin | 72277 | 0 | 306.271 | -0.203 | -2.298 | 21.067 | 7.627 | 36.486 | 1 |
| Amyrin | 73145 | 5 | 426.724 | 7.056 | -8.121 | 4470.509 | 2496.37 | 100 | 1 |
| Pinoresinol | 73399 | 0 | 358.39 | 2.849 | -4.05 | 1389.702 | 706.043 | 100 | 0 |
| Diosgenin | 99474 | 3 | 414.627 | 6.083 | -7.329 | 3439.889 | 1880.576 | 100 | 1 |
| Marmesin | 334704 | 0 | 246.262 | 2.052 | -3.092 | 984.51 | 486.432 | 92.532 | 0 |
| Gingerol | 442793 | 0 | 294.39 | 3.677 | -4.591 | 665.247 | 318.431 | 100 | 0 |
| Arachidonic Acid | 444899 | 1 | 304.472 | 5.85 | -4.167 | 754.779 | 464.222 | 100 | 1 |
| Oleic Acid | 445639 | 2 | 282.465 | 5.467 | -4.696 | 235.576 | 131.867 | 88.457 | 1 |
| Ferulic Acid | 445858 | 0 | 194.187 | 1.398 | -1.724 | 77.348 | 39.567 | 68.932 | 0 |
| Geraniol | 637566 | 3 | 154.252 | 2.595 | -2.728 | 2877.739 | 1550.709 | 100 | 0 |
| Bakuchicin | 3083848 | 0 | 186.167 | 0.962 | -1.284 | 2058.172 | 1079.411 | 91.884 | 0 |
| Perilloside A | 3086657 | 1 | 314.378 | 0.395 | -2.72 | 222.566 | 97.506 | 71.274 | 0 |
| Quercetin | 5280343 | 0 | 302.24 | 0.362 | -2.83 | 20 | 7.21 | 52.348 | 0 |
| Coniferin | 5280372 | 0 | 342.345 | -0.518 | -1.756 | 75.244 | 30.196 | 57.5 | 0 |
| Apigenin | 5280443 | 0 | 270.241 | 1.624 | -3.317 | 124.496 | 52.038 | 73.955 | 0 |
| Luteolin | 5280445 | 0 | 286.24 | 0.941 | -3.039 | 45.023 | 17.333 | 62.05 | 0 |
| Stigmasterol | 5280794 | 6 | 412.698 | 7.396 | -8.334 | 3439.965 | 1880.622 | 100 | 1 |
| Isoquercitrin | 5280804 | 4 | 464.382 | -1.37 | -2.483 | 3.216 | 1 | 2.086 | 2 |
| Kaempferol | 5280863 | 0 | 286.24 | 1.036 | -3.09 | 55.32 | 21.655 | 64.205 | 0 |
| Alpha-Linolenic Acid | 5280934 | 1 | 278.434 | 5.286 | -3.901 | 550.729 | 330.196 | 93.996 | 1 |
| Genistein | 5280961 | 0 | 270.241 | 1.694 | -3.039 | 170.821 | 73.252 | 76.823 | 0 |
| Mangiferin | 5281647 | 5 | 422.345 | -1.818 | -2.539 | 2.818 | 0.867 | 0 | 2 |
| Myricetin | 5281672 | 1 | 318.239 | -0.303 | -2.593 | 7.229 | 2.4 | 27.586 | 1 |
| Daidzein | 5281708 | 0 | 254.242 | 1.792 | -3.016 | 382.701 | 175.175 | 83.667 | 0 |
| Rosmarinic Acid | 5281792 | 1 | 360.32 | 1.195 | -3.324 | 2.141 | 0.819 | 39.86 | 0 |
| Shogaol | 5281794 | 0 | 276.375 | 3.429 | -2.864 | 1636.54 | 842.519 | 100 | 0 |
| Wedelolactone | 5281813 | 0 | 314.251 | 0.826 | -2.776 | 107.684 | 44.486 | 68.156 | 0 |
| Ellagic Acid | 5281855 | 0 | 302.197 | -1.306 | -1.912 | 7.958 | 2.663 | 35.42 | 0 |
| Astragalin | 5282102 | 1 | 448.382 | -0.765 | -2.651 | 8.468 | 2.848 | 13.152 | 2 |
| Bakuchiol | 5468522 | 2 | 256.387 | 5.161 | -5.534 | 3011.594 | 1628.817 | 100 | 1 |
| Corylifolin | 5470819 | 2 | 188.269 | 3.52 | -3.718 | 3011.384 | 1628.694 | 100 | 0 |
| beta-Glucan | 46173706 | 12 | 504.441 | -5.814 | -0.784 | 1.059 | 0.301 | 0 | 3 |
